# Regioselective palladium-catalyzed ring-opening reactions of C1-substituted oxabicyclo[2,2,1]hepta-2,5-diene-2,3-dicarboxylates

**DOI:** 10.3762/bjoc.12.25

**Published:** 2016-02-09

**Authors:** Michael Edmunds, Mohammed Abdul Raheem, Rebecca Boutin, Katrina Tait, William Tam

**Affiliations:** 1 Guelph-Waterloo Centre for Graduate Work in Chemistry and Biochemistry, Department of Chemistry and Biochemistry, University of Guelph, Guelph, Ontario, N1G 2W1, Canada

**Keywords:** aryl iodides, oxabenzonorbornadiene, palladium catalysis, regioselectivity, ring-opening reactions

## Abstract

Palladium-catalyzed ring-opening reactions of C1 substituted 7-oxanorbornadiene derivatives with aryl iodides were investigated. The optimal conditions for this reaction were found to be PdCl_2_(PPh_3_)_2_, ZnCl_2_, Et_3_N and Zn in THF. Both steric and electronic factors played a role in the outcome of the reaction as increasing the steric bulk on the bridgehead carbon decreased the yield. These reactions were found to be highly regioselective, giving only one of the two possible regioisomers in all cases. A diverse collection of novel, highly substituted biphenyl derivatives were obtained.

## Introduction

Palladium-catalyzed ring-opening reactions of 7-oxanorbornadiene with aryl iodides produce unsymmetrical, highly-substituted biphenyl derivatives making this reaction a very useful tool in organic synthesis [1–6]. The products of these reactions are also phthalates which have been used as intermediates in the synthesis of pharmaceuticals and biologically active agents, such as the anthracyclinone daunomycinone [7]. Phthalates are also used in paints, cosmetics, and as plasticizers in polymeric materials [8].

**Scheme 1:**
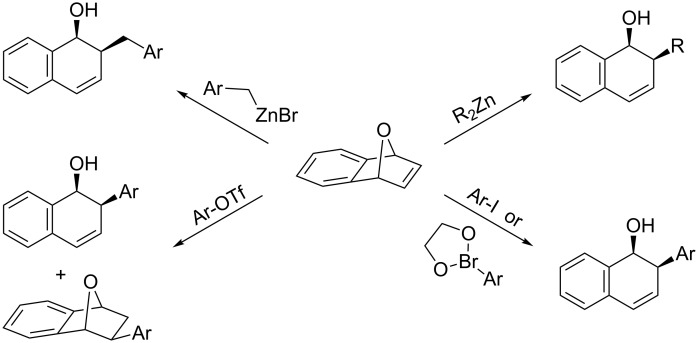
Palladium-catalyzed ring-opening reactions of oxabenzonorbornadiene.

**Scheme 2:**
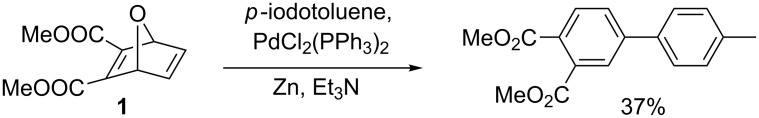
Palladium-catalyzed ring-opening of **1** with *p*-iodotoluene.

**Scheme 3:**
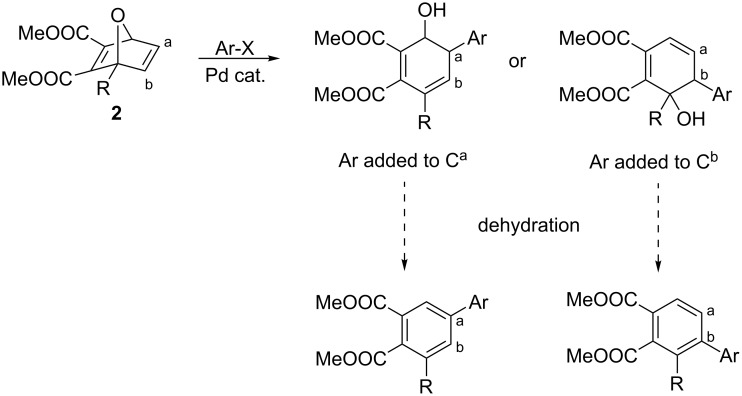
Potential regioisomers from the palladium-catalyzed ring-opening reaction of **2** with aryl iodides.

Although there are several examples of palladium-catalyzed ring-opening reactions of oxabenzonorbornadiene (Scheme 1), there is relatively little literature regarding reactions of non-aromatic 7-oxanorbornadiene systems [9–13]. To our knowledge, there has only been one example of **1** undergoing a ring-opening reaction, which used *p*-iodotoluene and a palladium catalyst (Scheme 2) [14]. The result was the addition of the aryl group to the unsubstituted double bond followed by dehydration to give an unsymmetrical biphenyl derivative. However, there have not been any investigations into the effect C1 substitution of **1** would have on the reaction. The addition of a substituent at the bridgehead carbon renders the bicyclic structure unsymmetrical and alters the steric and electronic factors imposed on the incoming palladium–aryl complex, which could affect the reactivity and regioselectivity of the reaction. Two regioisomers are possible for this reaction based on whether the aryl group is added to carbon a or carbon b of **2** (Scheme 3).

**Scheme 4:**
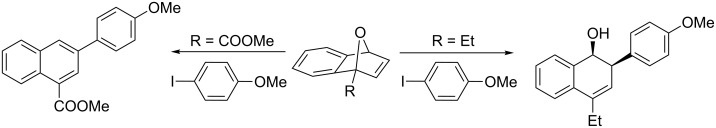
Palladium-catalyzed ring-opening of C1 substituted oxabenzonorbornadiene.

Our group recently investigated the effect of C1 substitution, with ethyl and methoxycarbonyl substituents, on the palladium-catalyzed ring-opening reaction of oxabenzonorbornadiene with aryl iodides (Scheme 4) [15]. We found that the electron-withdrawing methoxycarbonyl substituent on the aryl iodide or bridgehead carbon lowered the yield in all cases and gave aromatized products, while the electron-donating ethyl group on the aryl iodide or bridgehead carbon increased the yield, relative to the unsubstituted parent compound, in all cases. Despite the differences in yield and in the electronic nature of the substituents, only a single regioisomer was obtained through the addition of the aryl group to the olefin carbon furthest from the C1 substituent.

In this paper, we wanted to apply the arylative ring-opening strategy described above to non-aromatic C1 substituted 7-oxanorbornadiene systems with the goal of understanding its impact on reactivity and regioselectivity. The reaction is expected to follow the same general mechanism as oxabenzonorbornadiene with the aryl group being added to the olefin carbon furthest from the C1 substituent followed by dehydration, producing a novel, highly-substituted, biphenyl derivative.

## Results and Discussion

The optimization study focused on the palladium catalyst, Lewis acid additive and solvent, as the requirement for Et_3_N, zinc and high temperatures had previously been determined in related reactions (Table 1) [15]. C1-methyl-substitued oxanorbornadiene **2a** was selected for the optimization study due to the ease at which it can be synthesized in larger quantities.

**Table 1:** Optimization of palladium catalyst, Lewis acid additive, and solvent.

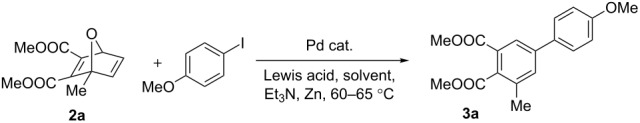					

Entry	Catalyst	Solvent	Lewis Acid	Time (h)	Yield (%)^a^

1	Pd(OAc)_2_	THF	ZnCl_2_	65	9^b^
2	Pd(OAc)_2_, Ph_3_P	THF	ZnCl_2_	17	48^b^
3	Pd(PPh_3_)_4_	THF	ZnCl_2_	19	58^b^
4	PdCl_2_(PPh_3_)_2_	THF	ZnCl_2_	16	88
5	PdCl_2_(PPh_3_)_2_	hexanes	ZnCl_2_	16	0
6	PdCl_2_(PPh_3_)_2_	methanol	ZnCl_2_	15	25
7	PdCl_2_(PPh_3_)_2_	DCM	ZnCl_2_	16	61
8	PdCl_2_(PPh_3_)_2_	toluene	ZnCl_2_	22	73
9	PdCl_2_(PPh_3_)_2_	DMF	ZnCl_2_	15	85
10	PdCl_2_(PPh_3_)_2_	THF	FeCl_3_	16	22
11	PdCl_2_(PPh_3_)_2_	THF	AlCl_3_	16	29
12	PdCl_2_(PPh_3_)_2_	THF	ZrCl_4_	16	47
13	PdCl_2_(PPh_3_)_2_	THF	ZnI_2_	16	48
14	PdCl_2_(PPh_3_)_2_	THF	CuCl_2_	18	85

^a^Isolated yield after column chromatography. **^b^**Yield based on ^1^H NMR using toluene as internal standard.

The palladium catalysts were selected based on their oxidation state and the presence or absence of phosphine ligands (Table 1, entries 1–4). The catalyst lacking a phosphine ligand gave a low yield and the reaction proceeded very slowly (9%, 65 h, Table 1, entry 1). The addition of phosphine exogenously greatly increased the speed and the yield of the reaction (48%, 17 h, Table 1, entry 2). The palladium catalyst, Pd(PPh_3_)_4_, gave a moderate yield (58%, 19 h, Table 1, entry 3), while the palladium(II) catalyst, PdCl_2_(PPh_3_)_2_, gave the best yield of the ring opened product and also reacted the fastest (88%, 16 h, Table 1, entry 4).

A variety of solvents were screened including polar aprotic, polar protic and nonpolar solvents (Table 1, entries 5–9). The nonpolar solvents (hexanes, dichloromethane, and toluene) resulted in a range of yields. There was no conversion to the ring opened product when the reaction was performed in hexanes (Table 1, entry 5), however, DCM and toluene gave much better yields at 61% and 73% respectively (Table 1, entries 7 and 8). The reaction using the polar protic solvent, methanol, gave a low yield (25%, Table 1, entry 6), while the polar aprotic solvents, DMF and THF, gave the best results at 85% and 88% respectively (Table 1, entries 9 and 4).

The Lewis acid additives gave a large range of yields (Table 1, entries 10–14). Iron and aluminium chlorides gave low yields (22%, Table 1, entry 10 and 29%, entry 11), zirconium chloride and zinc iodide gave moderate yields (47%, Table 1, entry 12 and 48%, entry 13) and copper chloride and zinc chloride gave the highest yields (85%, Table 1, entry 14 and 88%, entry 4). The combination of PdCl_2_(PPh_3_)_2_, THF, and zinc chloride resulted in the highest conversion to the ring-opened product.

Once the optimized conditions were found, **2a** was reacted with a variety of substituted aryl iodides to illustrate the scope of the reaction with different aryl iodides (Table 2). The effect of the relative position of the substituent to the iodide and the electronic nature of the substituents were investigated. The electron-donating groups, OMe and Me, gave the highest yield in the *para* position while the electron-withdrawing group, NO_2_, favoured ring-opening in the *meta* position. The highest yield for the electron-donating groups was obtained with *p*-iodoanisole (88%, Table 2, entry 1). Moderate yields were obtained with *m-* and *o*-iodoanisoles (62%, Table 2, entry 2 and 69%, entry 3). *p*-Iodotoluene also gave a high yield (82%, Table 2, entry 4). The yield for *m*-iodotoluene was close (79%, Table 2, entry 5) but there was a dramatic drop in the yield for *o*-iodotoluene (30%, Table 2, entry 6), potentially as a result of steric interactions with *ortho* substituents at site of Pd insertion.

**Table 2:** Effect of aryl iodide substitution on the ring-opening of oxanorbornadiene.

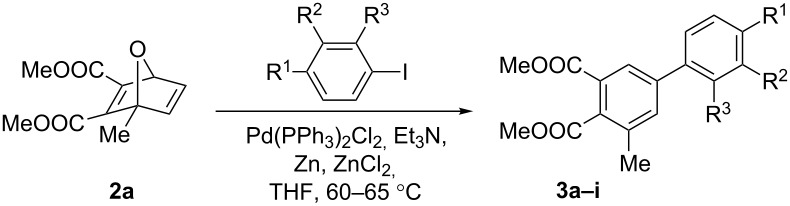						

Entry	R^1^	R^2^	R^3^	Time (h)	Product	Yield (%)^a^

1	OMe	H	H	16	**3a**	88
2	H	OMe	H	17	**3b**	62
3	H	H	OMe	17	**3c**	69
4	Me	H	H	16	**3d**	82
5	H	Me	H	16	**3e**	79
6	H	H	Me	19	**3f**	30
7	NO_2_	H	H	16	**3g**	73
8	H	NO_2_	H	16	**3h**	88
9	H	H	NO_2_	19	**3i**	47

^a^Isolated yield after column chromatography.

The trend seen with electron-donating substituents on the aryl iodide was not seen with the electron-withdrawing substituent, NO_2_; *p-*iodonitrobenzene did not give the highest yield for the ring-opened product (73%, Table 2, entry 7). Instead, the highest yield was seen with *m*-iodonitrobenzene (88%, Table 2, entry 8); the same yield as was seen with *p*-iodoanisole, and the lowest yield was seen with *o*-iodonitrobenzene (47%, Table 2, entry 9).

The effect of substitution on the bridgehead carbon of **1** was investigated to determine its impact on the outcome of the ring-opening reaction (Table 3). A wide variety of substituents were chosen, including electron-withdrawing and donating groups, bulky groups, and aromatic rings, in order to examine the impact of steric and electronic changes at the C1 position. The greatest yield for the ring-opened derivative was obtained with the methyl substituent (88%, Table 3, entry 1). When the size of the substituent was increased by one carbon, an ethyl group, the yield decreased to 69% (Table 3, entry 2) and when an even bulkier phenyl group was substituted at the C1 position, the yield was reduced to 33% (Table 3, entry 3). Increasing the steric bulk on the bridgehead carbon appears to impact the reactions’ outcome by hindering access to the site of carbo-palladation. SiMe_3_ gave a moderate yield of 64% (Table 3, entry 4). The silicon–carbon bond is longer than carbon–carbon bonds, making TMS less bulky than its hydrocarbon counterparts. Both of the electron-withdrawing functional groups, methyl ester, and acetyl, resulted in 0% conversion to the ring-opened product (Table 3, entries 5 and 6). Based on these results, it appears that both electronic and steric interactions at the bridgehead carbon play an important role in the outcome of the reaction.

**Table 3:** Effect of C1 substitution on the ring-opening reaction of oxanorbornadiene.

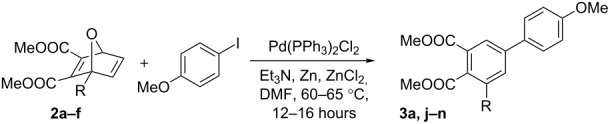				

Entry	OBD	R	Product	Yield (%)^a^

1	**2a**	Me	**3a**	88
2	**2b**	Et	**3j**	69
3	**2c**	Ph	**3k**	33
4	**2d**	SiMe_3_	**3l**	64
5	**2e**	COOMe	**3m**	0
6	**2f**	COCH_3_	**3n**	0

^a^Isolated yield after column chromatography.

**Scheme 5:**
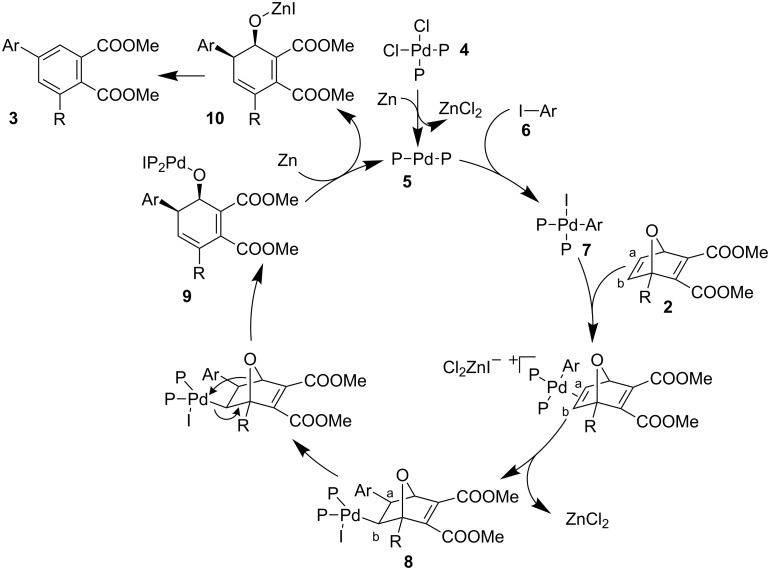
Proposed mechanism for the palladium-catalyzed ring-opening reaction of oxanorbornadiene.

A mechanism for the ring-opening reaction of **2** with aryl iodides has been proposed based on the results obtained (Scheme 5). The reaction begins with the reduction of the palladium(II) catalyst, **4**, to palladium(0) **5** by zinc. The Pd(0) catalyst complexes with the aryl iodide **6** forming the palladium-aryl complex **7**. This complex undergoes *exo* selective coordination with **2**, which is likely promoted by the Lewis acid ZnCl_2_ removing the iodide from **7** allowing it to associate with the oxanorbornadiene. This is quickly followed by carbopalladation onto the exposed olefin to give intermediate **8**. There are two possible regioisomers, depending on whether the aryl group adds to C^a^ or C^b^. In all cases, only one regioisomer was seen as a result of the addition of the aryl group to C^a^. Cleavage of the β-oxygen gives the ring-opened intermediate **9**. The palladium species is reduced, producing the final intermediate **10** and regenerating the catalyst. Compound **10** rapidly undergoes base-catalyzed dehydration to give the final product **3**.

## Conclusion

In conclusion, we have demonstrated the first examples of palladium-catalyzed ring-opening reactions of **1** with aryl iodides. This reaction was highly regioselective giving only one of the two possible regioisomers via the addition of the aryl group to the less hindered carbon of the olefin (C^a^), followed by dehydration, producing novel highly-substituted biphenyl derivatives. We investigated the efficacy of various ring-opening conditions and found that PdCl_2_(PPh_3_)_2_, THF, ZnCl_2_, Et_3_N, high temperature, and Zn gave the highest yield.

## Supporting Information

File 1 Experimental procedures and analytical data.

File 2 Copies of ^1^H and ^13^C NMR spectra for compounds **3a–l**.
