# Supplementary material for: Regioselective palladium-catalyzed ring-opening reactions of C1-substituted oxabicyclo[2,2,1]hepta-2,5-diene-2,3-dicarboxylates
Source: Beilstein J Org Chem. 2016 Feb 9;12:239–44. doi: 10.3762/bjoc.12.25 (PMC4778517; doi:10.3762/bjoc.12.25)
Supplement: File 1 — Experimental procedures and analytical data. [file Beilstein_J_Org_Chem-12-239-s001.pdf]

# Supporting Information

# for

# Regioselective palladium-catalyzed ring-opening reactions of C1-substituted oxabicyclo[2,2,1]hepta-2,5-diene-2,3-dicarboxylates

Michael Edmunds, Mohammed Abdul Raheem, Rebecca Boutin, Katrina Tait, and William Tam*

Address: Guelph-Waterloo Centre for Graduate Work in Chemistry and Biochemistry, Department of Chemistry and Biochemistry, University of Guelph, Guelph, Ontario, N1G 2W1, Canada

Email: William Tam - wtam@uoguelph.ca

*Corresponding author

## Experimental procedures and analytical data

### Table of Contents

General considerations S2

Previously reported key compounds **2a–f** S2

General procedure for ring opening reactions of $C_1$ substituted oxabenzonorbornadienes S2–S3

Characterization data for compounds **3a–l** S3–S7

**General considerations**

All ring opening reactions were carried out under inert atmospheric conditions. All glassware was oven-dried overnight before use. Flash column chromatography was performed on 230–400 mesh silica gel purchased from Silicycle. Analytical TLC was performed on pre-coated silica gel 250 μm 60 F254 aluminum plates purchased from Silicycle. TLC visualization was carried out under UV light and *p*-anisaldehyde stain. $^{1}$H and $^{13}$C NMR spectra were recorded on a Bruker Avance 400 MHz spectrometer ($CDCl_3$: δ 7.24 ppm ($^{1}$H at 400 MHz) or δ 77.0 ppm ($^{13}$C at 100 MHz)). HRMS analyses were performed at the Queen's Mass Spectrometry and Proteomics Unit, Kingston, Ontario. The samples were ionized by electron impact (EI) or electrospray ionization (ESI) as specified and detection of the ions was performed by time of flight (TOF).

**Experimental procedures and full characterization data for previously reported key compounds.**

For complete experimental procedures and full characterization data of all starting materials **2a–f**, see our previous report on the synthesis of $C_1$-substituted 7-oxabenzonorbornadienes [16].

**General procedure for palladium-catalyzed ring-opening reactions of $C_1$-substituted oxabenzonorbornadienes with aryl iodides (Table 2 and Table 3).**

In a similar manner to the experimental procedure described in [15], $PdCl_2(PPh_3)_2$ (0.016 g, 0.05 mmol), zinc powder (0.315 g, 5 mmol), $C_1$-substituted oxabenzonorbornadiene derivative (0.50 mmol) and zinc chloride (0.025 mmol) were weighed out in a dry flask equipped with a magnetic stirring bar. After the flask was sealed with a rubber septum the system was evacuated and

purged with nitrogen gas three times followed by addition of the solvent (3.0 mL). The flask was charged with triethylamine (4.0 mmol) and aryl halide (0.55 mmol) via a syringe through the rubber septum into the flask. The mixture was heated with stirring at 60–65 °C until the bicyclic alkene derivative was consumed as indicated by TLC analysis of the solution.

The reaction mixture was then cooled and stirred under air for 15-20 minutes at room temperature. The reaction mixture was diluted with dichloromethane (10–15 mL) and filtered through a pad of Celite™. The obtained filtrate was concentrated on a rotary evaporator to obtain a brown oily residue. The crude residue was purified by flash chromatography, eluting with mixtures of EtOAc/hexanes to provide the product.

**Dimethyl 4′-methoxy-1,1′-biphenyl-5-methyl-3,4-dicarboxylate, 3a (Table 2, entry 1):** Yield: 88% (113 mg, 0.5 mmol) $R_f$ = 0.25 (EtOAc–hexanes, 1:5); IR (ν, $cm^{-1}$); 3055, 2987, 2954, 1731, 1609, 1517, 1438, 1333, 1266, 1106, 1069, 1033; $^1$H NMR ($CDCl_3$, 300 MHz) δ: 2.37 (s, 3H), 3.80 (s, 3H), 3.88 (s, 3H), 3.93 (s, 3H), 6.95 (dd, J=6.7 & 2.1 Hz, 2H), 7.50 (dd, J = 6.7 & 2.1 Hz, 2H), 7.54 (d, J = 1.7 Hz, 1H), 7.97 (d, J=1.7 Hz, 1H); $^{13}$C NMR ($CDCl_3$, 75 MHz) δ: 19.2, 52.4, 55.2, 114.3, 125.5, 128.1 ; 128.5, 131.6, 132.2, 133.1; 136.0, 141.6, 159.7, 166.3, 169.7; HRMS (ESI) calcd. for $C_{18}H_{19}O_5$ ($M+H^+$): 315.1232; found: 315.1236.

**Dimethyl 3′-methoxy-1,1′-biphenyl-5-methyl-3,4-dicarboxylate, 3b (Table 2, entry 2):** Yield: 62% (54mg, 0.17 mmol); $R_f$ = 0.38 (EtOAc-Hexanes, 1:3); IR (ν, $cm^{-1}$): 2952, 1728, 1601, 1438, 1334, 1266, 1216, 1195, 1180, 1160, 1103, 1068, 1049, 789, 772, 738, 699. $^1$H NMR (400 MHz, $CDCl_3$) δ: 2.44 (s, 3H), 3.90 (s, 3H), 3.94 (s, 3H), 3.99 (s, 3H), 6.94-6.97 (m, 1H), 7.13-7.14 (m, 1H), 7.19-7.21 (m, 1H), 7.40 (t, J = 7.9 Hz, 1H), 7.63-7.64 (m, 1H), 8.06-8.07 (m, 1H). $^{13}$C NMR (100 MHz, $CDCl_3$) δ: 19.3, 52.6, 55.4, 113.0, 113.28, 119.7, 126.2, 128.4, 130.0, 133.0,

134.1, 134.5, 136.2, 140.8, 142.0, 160.0, 166.3, 169.9; HRMS (EI) calcd for $C_{18}H_{18}O_5$ ($M^+$): 314.1154; found: 314.1148.

**Dimethyl 2′-methoxy-1,1′-biphenyl-5-methyl-3,4-dicarboxylate, 3c (Table 2, entry 3):** Yield: 69% (51.6mg, 0.16 mmol); $R_f$ = 0.34 (EtOH-Hexanes, 1:3); IR (ν, $cm^{-1}$): 2952, 1728, 1498, 1438, 1331, 1268, 1251, 1193, 1161, 1097, 1069, 1025, 756, 738. $^1H$ NMR (400 MHz, $CDCl_3$) δ: 2.42 (s, 3H), 3.83 (s, 3H), 3.92 (s, 3H), 3.98 (s, 3H), 7.00-7.08 (m, 2H), 7.28-7.32 (m, 1H), 7.36-7.40 (m, 1H), 7.58-7.59 (m, 1H). $^{13}C$ NMR (100 MHz, $CDCl_3$) δ: 19.2, 52.6, 55.6, 111.3, 120.9, 127.6, 128.7, 128.9, 129.5, 130.7, 133.6, 135.2, 135.6, 139.7, 156.4, 166.5, 170.1; HRMS (EI) calcd for $C_{18}H_{18}O_5$ ($M^+$): 314.1154; found: 314.1160.

**Dimethyl 4′-methyl-1,1′-biphenyl-5-methyl-3,4-dicarboxylate, 3d (Table 2, entry 4):** Yield: 82% (72mg, 0.24 mmol); $R_f$ = 0.42 (EtOAc-Hexanes, 1:3); IR (ν, $cm^{-1}$): 2951, 1722, 1607, 1518, 1437, 1335, 1254, 1214, 1196, 1162, 1105, 1069, 1018, 899, 822, 790, 765, 737. $^1H$ NMR (400 MHz, $CDCl_3$) δ: 2.43 (s, 3H), 2.44 (s, 3H), 3.94 (s, 3H), 3.99 (s, 3H), 7.28-7.30 (m, 2H), 7.51 (s, 1H), 7.53 (s, 1H), 7.63 (d, J = 1.0 Hz, 1H), 8.06 (d, J = 1.5 Hz, 1H). $^{13}C$ NMR (100 MHz, $CDCl_3$) δ: 19.3, 21.2, 52.6, 126.0, 127.0, 128.4, 129.7, 132.7, 133.6, 136.1, 136.4, 138.1, 142.1, 166.4, 170.0. HRMS (EI) calcd for $C_{18}H_{18}O_4$ ($M^+$): 298.1205; found: 298.1201.

**Dimethyl 3′-methyl-1,1′-biphenyl-5-methyl-3,4-dicarboxylate, 3e (Table 2, entry 5):** Yield: 79% (57.8 mg, 0.19 mmol); $R_f$ = 0.41(EtOAc-Hexanes, 1:3); IR (ν, $cm^{-1}$): 2951, 1728, 1606, 1437, 1333, 1272, 1201, 1160, 1105, 1072, 787, 739. $^1H$ NMR (400 MHz, $CDCl_3$) δ: 2.45 (s, 3H), 2.46 (s, 3H), 3.94 (s, 3H), 3.99 (s, 3H), 7.22-7.28 (m, 1H), 7.35-7.43 (m, 3H), 7.64 (d, J = 1.1 Hz, 1H), 8.06 (d, J = 1.4 Hz, 1H). $^{13}C$ NMR (100 MHz, $CDCl_3$) δ: 19.3, 21.5, 52.6, 124.3,

126.2, 127.5, 128.1, 128.4, 128.5, 128.9, 129.1, 133.0, 136.1, 138.6, 139.3, 142.3, 166.4, 169.9. HRMS (EI) calcd for $C_{18}H_{18}O_4$ ($M^+$): 298.1205; found: 298.1198.

**Dimethyl 2′-methyl-1,1′-biphenyl-5-methyl-3,4-dicarboxylate, 3f (Table 2, entry 6):** Yield: 30% (40.3 mg, 0.14 mmol); $R_f$ = 0.50 (EtOAc-Hexanes, 1:3); IR (ν, $cm^{-1}$): 2952, 1731, 1437, 1331, 1268, 1247, 1198, 1160, 1096, 1069, 758. $^1$H NMR (400 MHz, $CDCl_3$) δ: 2.28 (s, 3H), 2.43 (s, 3H), 3.92 (s, 3H), 7.20-7.22 (m, 1H), 7.25-7.32 (m, 3H), 7.39-7.40 (m, 1H), 7.83-7.84 (m, 1H). $^{13}$C NMR (100 MHz, $CDCl_3$) δ: 19.2, 20.4, 52.6, 126.0, 127.7, 128.0, 128.3, 129.6, 129.8, 130.5, 133.7, 134.9, 135.2, 135.5, 140.1, 143.0, 166.4, 170.0. HRMS (EI) calcd for $C_{18}H_{18}O_4$ ($M^+$): 298.1205; found: 298.1211.

**Dimethyl 4′-nitro-1,1′-biphenyl-5-methyl-3,4-dicarboxylate, 3g (Table 2, entry 7):** Yield: 73% (47.7 mg, .14 mmol); $R_f$ = 0.31 (EtOAc-Hexanes 1:3); IR (ν, $cm^{-1}$): 3055, 2954, 1731, 1598, 1521, 1438, 1346, 1266, 1199, 1164, 1104, 1068, 898, 849, 791, 738, 705; $^1$H NMR (400 MHz, $CDCl_3$) δ: 2.47 (s, 3H), 3.96 (s, 3H), 4.00 (s, 3H), 7.67-7.68 (m, 1H), 7.77-7.79 (m, 2H), 8.10 (d, J = 1.8 Hz, 1H), 8.33-8.36 (m, 2H). $^{13}$C NMR (100 MHz, $CDCl_3$) δ: 19.3, 52.8, 52.8, 124.3, 126.5, 128.0, 128.8, 133.1, 135.5, 136.8, 139.7, 145.7, 147.6, 165.9, 169.4. HRMS (EI) calcd for $C_{18}H_{18}O_4$ ($M^+$): 329.0899; found: 329.0901.

**Dimethyl 3′-nitro-1,1′-biphenyl-5-methyl-3,4-dicarboxylate, 3h (Table 2, entry 8):** Yield: 88% (77.4 mg, 0.235 mmol); $R_f$ = 0.22 (EtOAc-Hexanes, 1:3); IR (ν, $cm^{-1}$): 2952, 1729, 1608, 1530, 1438, 1351, 1331, 1276, 1197, 1162, 1120, 1084, 1068, 812, 791, 740. $^1$H NMR (400 MHz, $CDCl_3$) δ: 2.46 (s, 3H), 3.95 (s, 3H), 4.00 (s, 3H), 7.65-7.69 (m, 2H), 7.94 (d, J = 7.8 Hz, 1H), 8.09 (m, 1H), 8.25-8.28 (m, 1H), 8.47-8.48 (m, 1H). $^{13}$C NMR (100 MHz, $CDCl_3$) δ: 19.3,

52.7, 52.8, 122.0, 122.9, 126.2, 128.8, 130.1, 132.9, 133.1, 135.2, 136.8, 139.5, 141.0, 148.7, 165.9, 169.4. HRMS (EI) calcd for $C_{18}H_{18}O_4$ ($M^+$): 329.0899; found: 329.0892.

**Dimethyl 2′-nitro-1,1′-biphenyl-5-methyl-3,4-dicarboxylate, 3i (Table 2, entry 9):** Yield: 47% (49.8 mg, 0.15 mmol); $R_f$ = 0.26 (EtOAc-Hexanes, 1:3); IR (ν, $cm^{-1}$): 2954, 1722, 1521, 1440, 1349, 1332, 1296, 1274, 1238, 1162, 1086, 744. $^1$H NMR (400 MHz, $CDCl_3$) δ: 2.40 (s, 3H), 3.91 (s, 3H), 4.00 (s, 3H), 7.36-7.37 (m, 1H), 7.41-7.43 (m, 1H), 7.55-7.59 (m, 1H), 7.66-7.70 (m, 1H), 7.82-7.83 (m, 1H), 7.97-7.99 (m, 1H). $^{13}$C NMR (100 MHz, $CDCl_3$) δ: 19.2, 52.6, 52.7, 124.5, 127.1, 128.2, 129.0, 132.0, 132.8, 133.7, 135.1, 136.2, 138.8, 148.7, 165.8, 169.6. HRMS (EI) calcd for $C_{18}H_{18}O_4$ ($M^+$): 329.0899; found: 329.0892.

**Dimethyl 4′-methoxy-1, 1′-biphenyl-5-ethyl-3, 4-dicarboxylate, 3j (Table 3, entry 2):** Yield: 69% (113 mg, 0.5 mmol); $R_f$ = 0.29 (EtOAc–Hexanes, 1:5); IR (ν, $cm^{-1}$); 3055, 2973, 2953, 1729, 1609, 1558, 1436, 1265, 1181, 1108, 1032 $cm^{-1}$; $^1$H NMR (300 MHz, $CDCl_3$): δ 1.26 (t, J= 7.6 Hz, 3H), 2.69 (q, J=7.6 Hz, 2H), 3.83 (s, 3H), 3.89 (s, 3H), 3.93 (s, 3H), 6.97 (dd, J=6.7 & 2.1 Hz, 2H), 7.53 (dd, J = 6.7 & 2.1 Hz, 2H), 7.59 (d, J = 1.8 Hz, 1H), 8.00 (d, J=1.8 Hz, 1H): $^{13}$C NMR (75 MHz, $CDCl_3$): 15.6, 26.5, 52.40, 52.44, 55.3, 114.3, 125.8, 128.2; 128.5, 131.0, 131.9, 132.7; 141.9, 142.3, 159.8, 166.4, 169.8; HRMS (ESI) calcd. for $C_{19}H_{21}O_5$ ($M+H^+$): 329.1389; found: 329.1392

**Dimethyl 4′-methoxy-1, 1′-biphenyl-5-phenyl-3, 4-dicarboxylate, 3k (Table 3, entry 3):** Yield: 33% (62 mg, 0.5 mmol); $R_f$ = 0.16 (EtOAc–Hexanes, 1:5); IR (ν, $cm^{-1}$); 3054, 2987, 1731, 1609, 1516, 1438, 1422, 1265, 1180, 1032, 735 $cm^{-1}$; $^1$H NMR (400 MHz, $CDCl_3$): δ 3.67 (s, 3H), 3.84 (s, 3H), 3.92 (s, 3H), 6.98 (d, J= 8.8 Hz, 2H), 7.32-7.42 (m, 5H), 7.57 (d, J = 8.8 Hz, 2H), 7.71 (d, J=1.9 Hz, 1H), 8.16 (d, J=1.9 Hz, 1H): $^{13}$C NMR (100 MHz, $CDCl_3$): 52.3, 52.6,

55.4, 114.4, 126.9, 127.9; 128.3, 128.6, 128.8, 131.4, 132.1, 132.7, 139.4; 141.1, 141.8, 159.9, 166.3, 169.3; HRMS (ESI) calcd. for $C_{23}H_{21}O_5$ (M+H$^+$): 377.1383; found: 377.1379.

**Dimethyl 4′-methoxy-1, 1′-biphenyl-5-trimethylsilyl-3, 4-dicarboxylate, 3l (Table 3, entry 4):** Yield: 64% (119 mg, 0.5 mmol); $R_f$ = 0.38 (EtOAc–Hexanes, 1:5); IR (ν, cm$^{-1}$); 3054, 2987, 1729, 1608, 1515, 1422, 1265, 1180, 1139, 896, 739 cm$^{-1}$; $^1$H NMR (400 MHz, $CDCl_3$): δ 0.33 (s, 9H), 3.83 (s, 3H), 3.89 (s, 6H), 6.98 (d, J= 8.7 Hz, 2H), 7.52 (d, J = 8.7 Hz, 2H), 7.88 (d, J=1.9 Hz, 1H), 8.06 (d, J=1.9 Hz, 1H): $^{13}$C NMR (100 MHz, $CDCl_3$): -0.4, 52.33, 52.51, 55.3, 114.4, 128.3, 128.4; 129.3, 132.0, 136.3, 137.8, 139.6; 141.2, 159.7, 167.1, 170.5; HRMS (ESI) calcd. for $C_{20}H_{25}O_5Si$ (M+H$^+$): 373.1471; found: 373.1468.
