# Supplementary material for: Regioselective palladium-catalyzed ring-opening reactions of C1-substituted oxabicyclo[2,2,1]hepta-2,5-diene-2,3-dicarboxylates
Source: Beilstein J Org Chem. 2016 Feb 9;12:239–44. doi: 10.3762/bjoc.12.25 (PMC4778517; doi:10.3762/bjoc.12.25)
Supplement: File 2 — Copies of 1H and 13C NMR spectra for compounds 3a–l. [file Beilstein_J_Org_Chem-12-239-s002.pdf]

Supporting Information
for
Regioselective palladium-catalyzed ring-opening reactions of C1-substituted oxabicyclo[2,2,1]hepta-2,5-diene-2,3-dicarboxylates

Michael Edmunds, Mohammed Abdul Raheem, Rebecca Boutin, Katrina Tait, and William Tam*

Address: Guelph-Waterloo Centre for Graduate Work in Chemistry and Biochemistry, Department of Chemistry and Biochemistry, University of Guelph, Guelph, Ontario, N1G 2W1, Canada

Email: William Tam - wtam@uoguelph.ca
*Corresponding author

Copies of ^{1}H and ^{13}C NMR spectra for compounds 3a–l

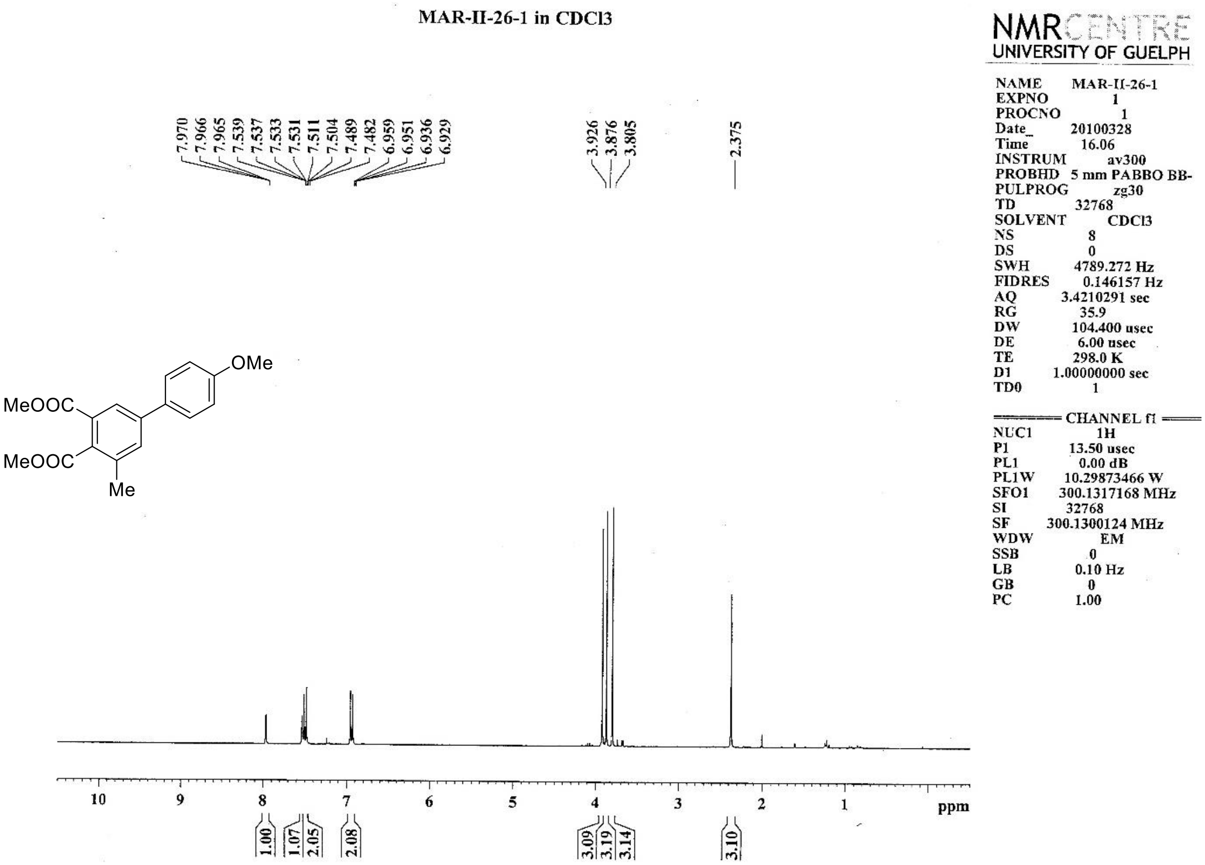

300 MHz ^{1}H NMR spectrum of **3a** in $CDCl_3$

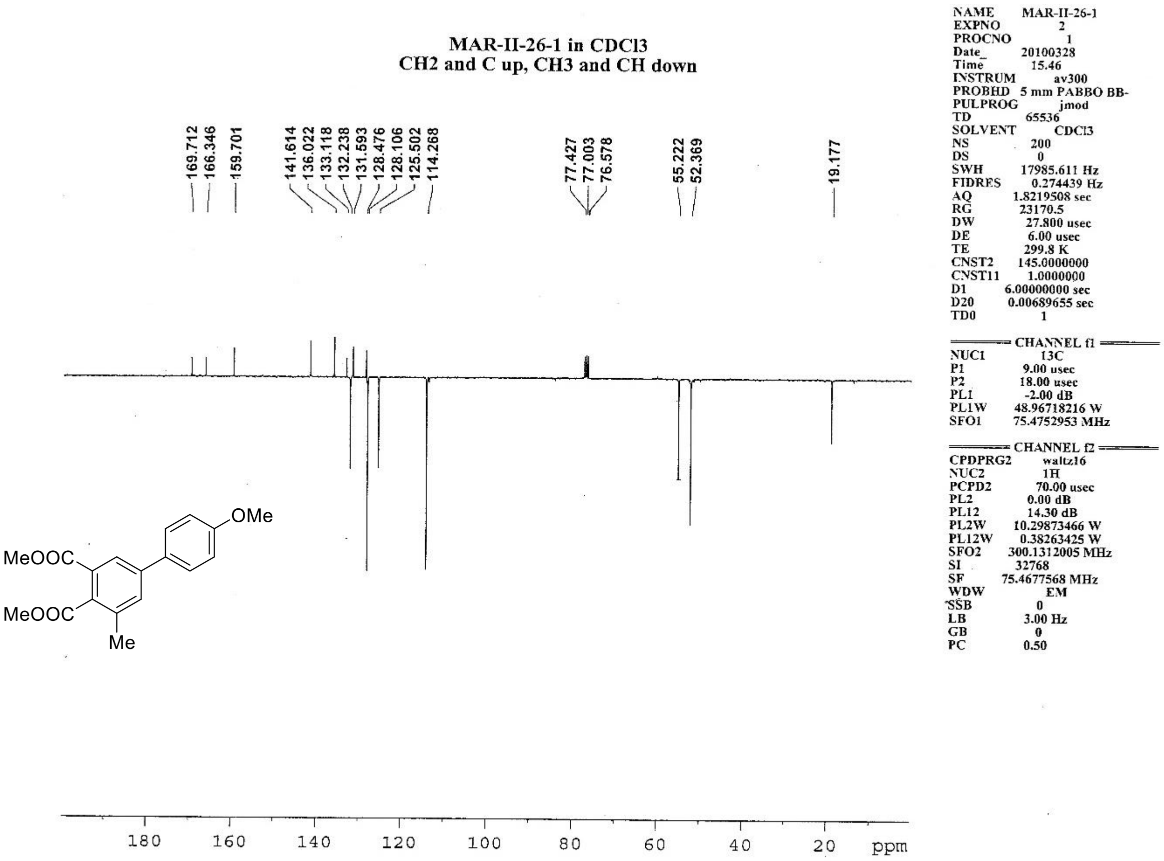

75 MHz ^{13}C NMR spectrum of **3a** in $CDCl_3$

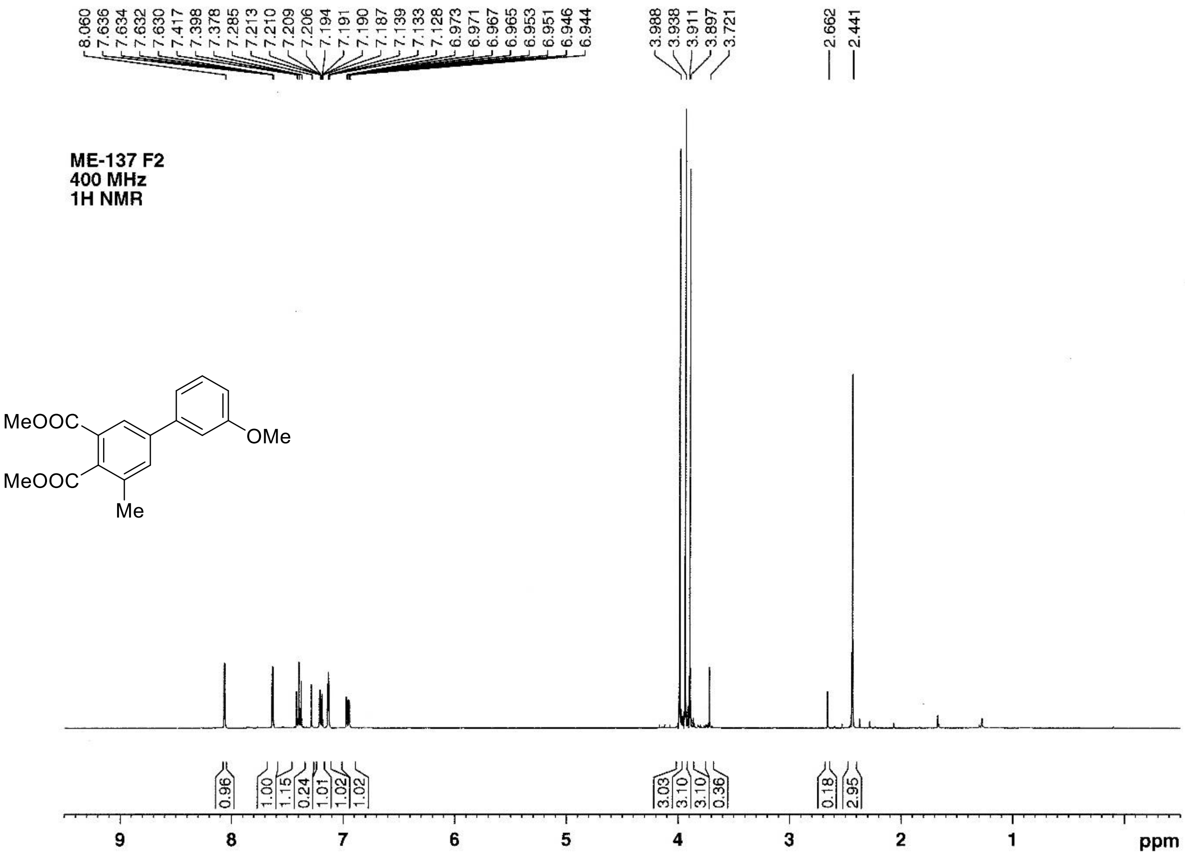

400 MHz ^{1}H NMR spectrum of 3**b** in $CDCl_3$

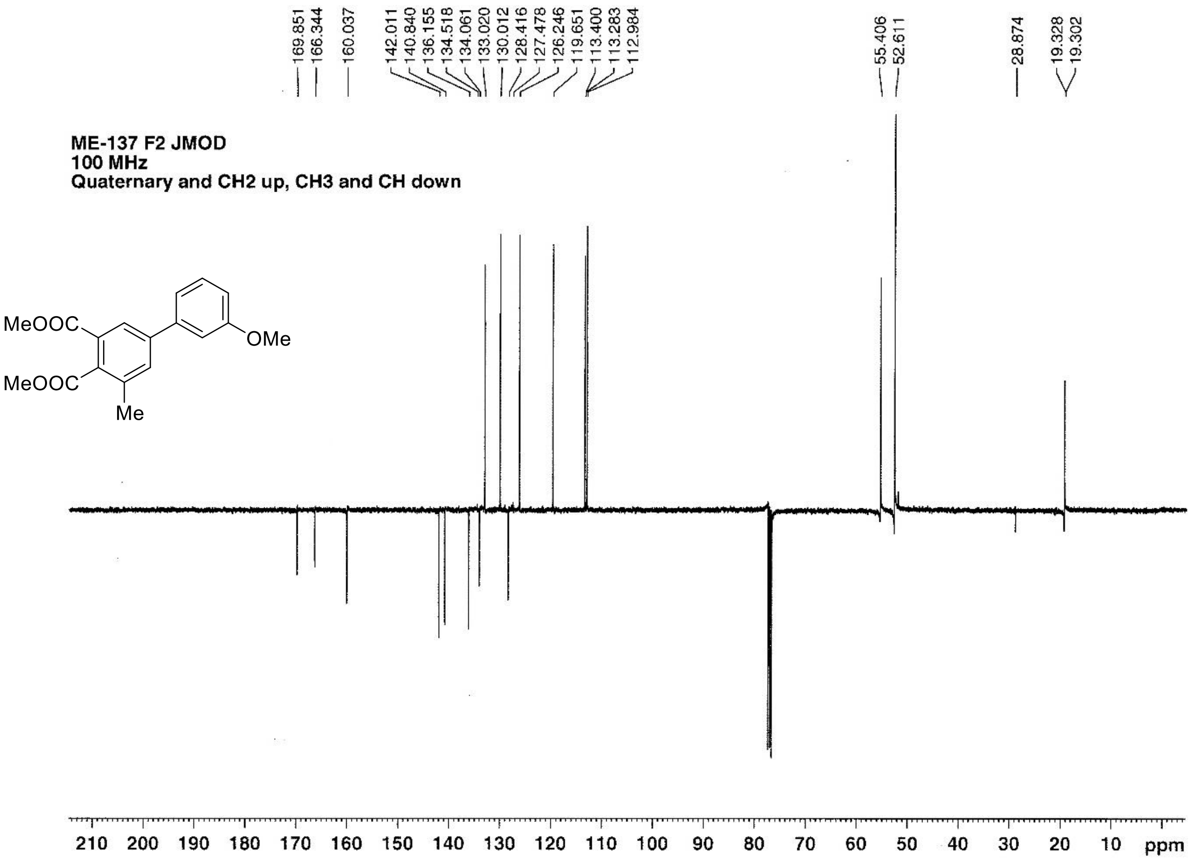

100 MHz ^{13}C NMR spectrum of 3**b** in $CDCl_3$

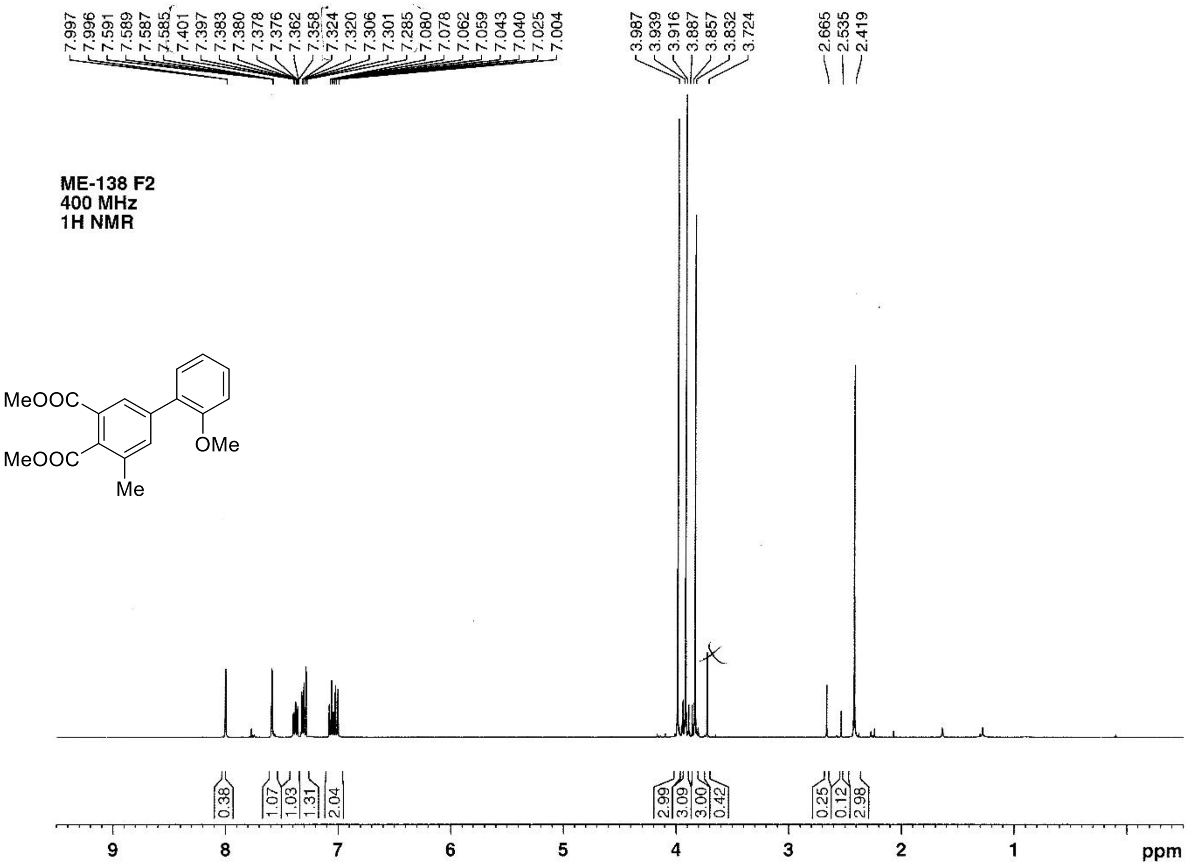

400 MHz ^{1}H NMR spectrum of **3c** in $CDCl_3$

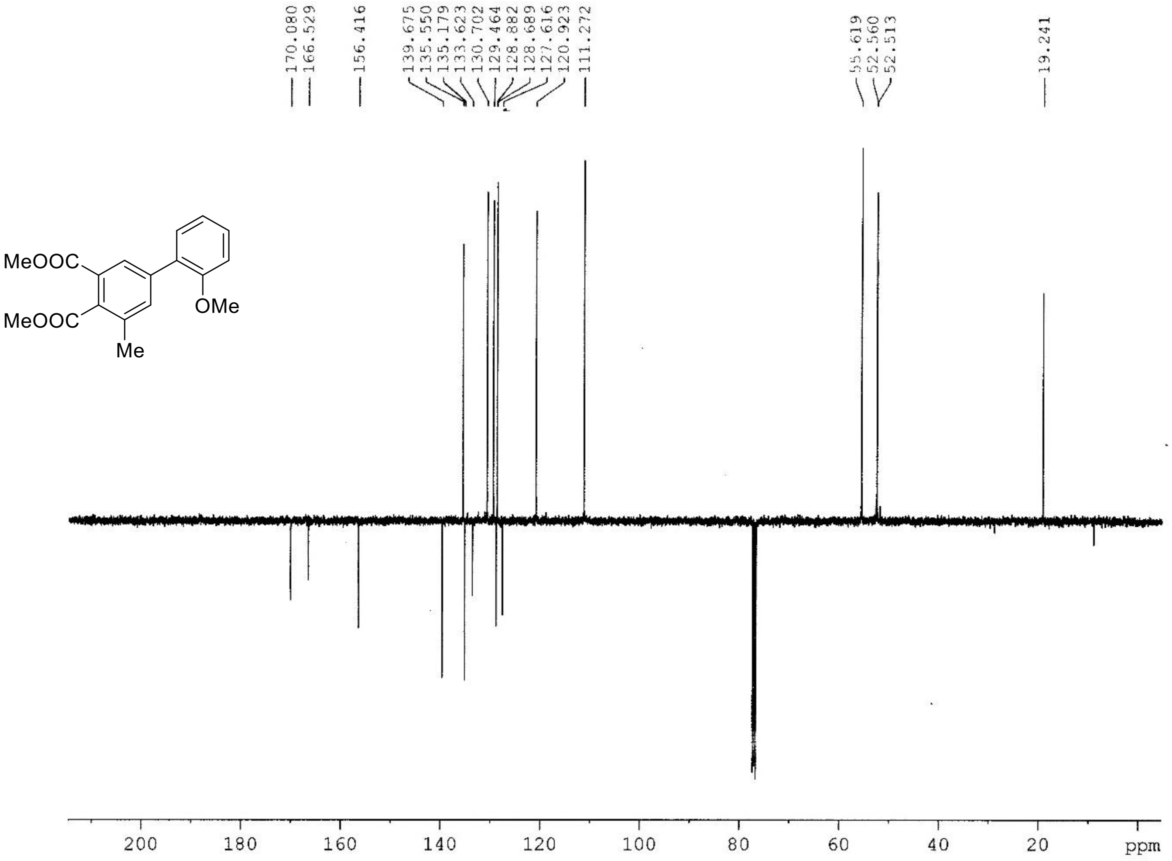

100 MHz ^{13}C NMR spectrum of **3c** in $CDCl_3$

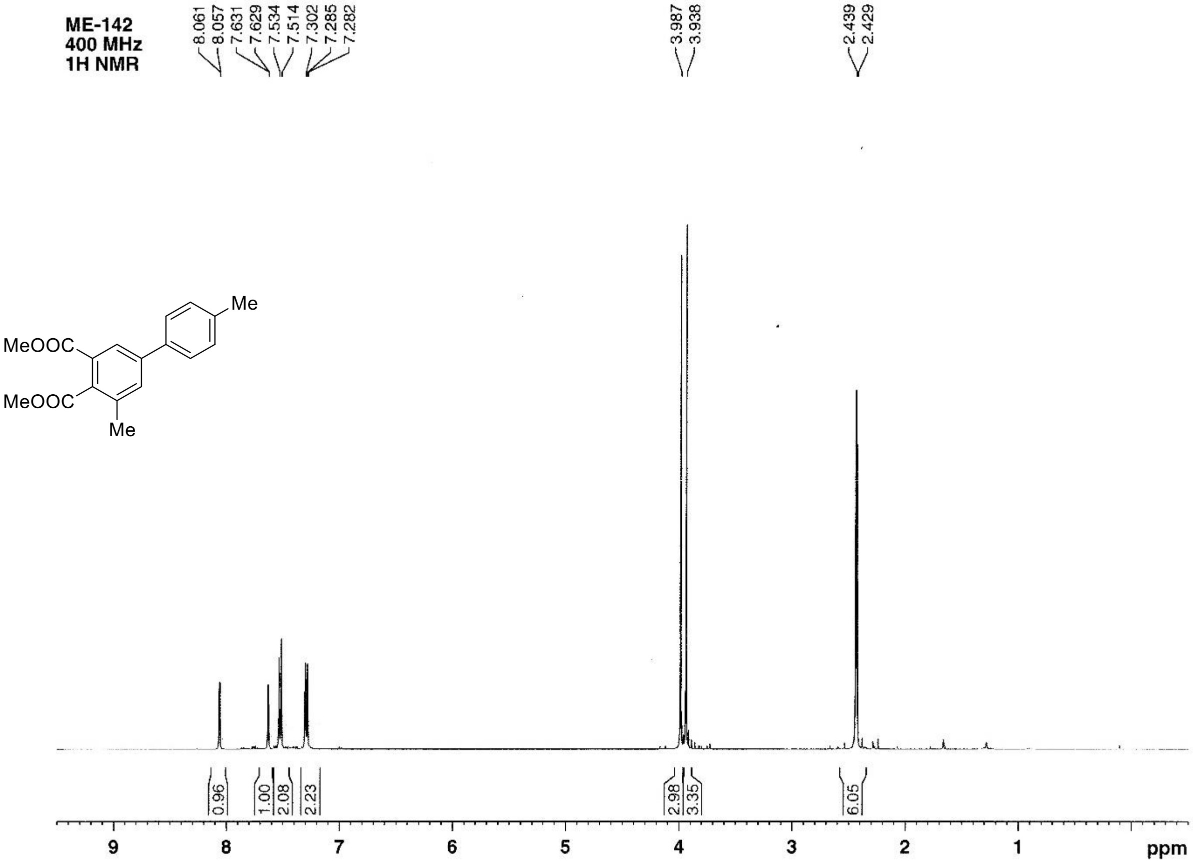

400 MHz ^{1}H NMR spectrum of **3d** in $CDCl_3$

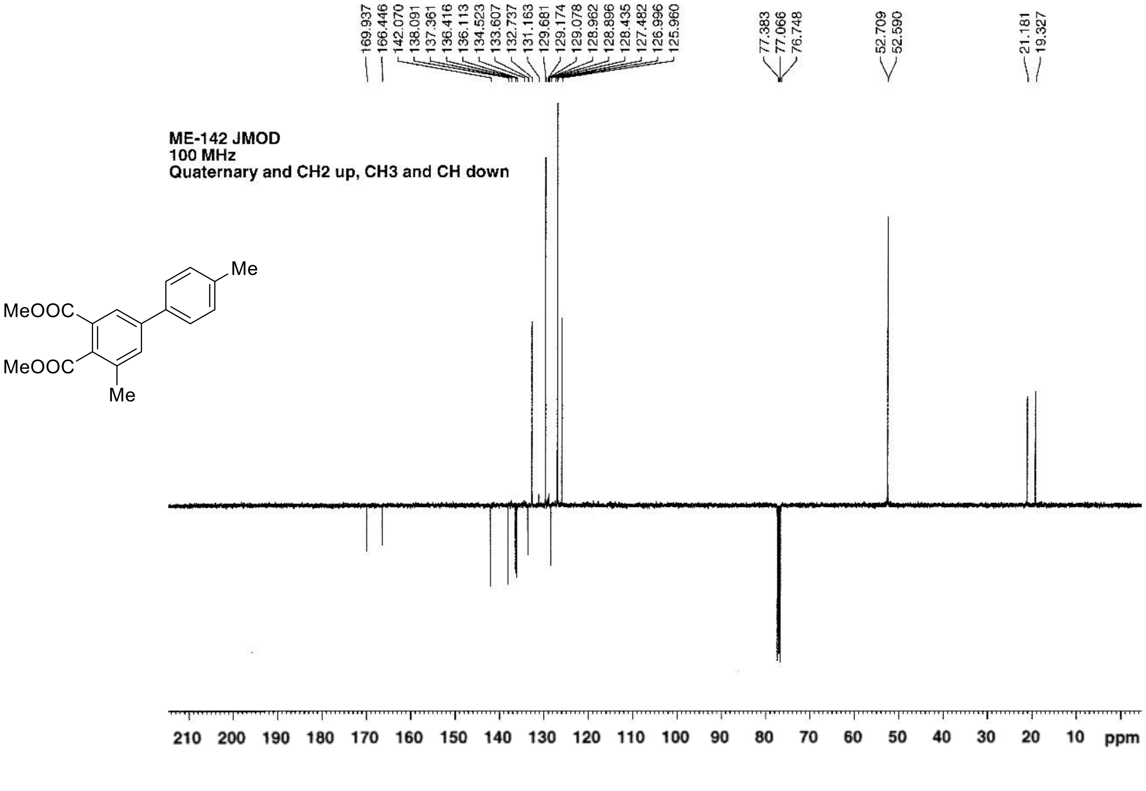

100 MHz ^{13}C NMR spectrum of **3d** in $CDCl_3$

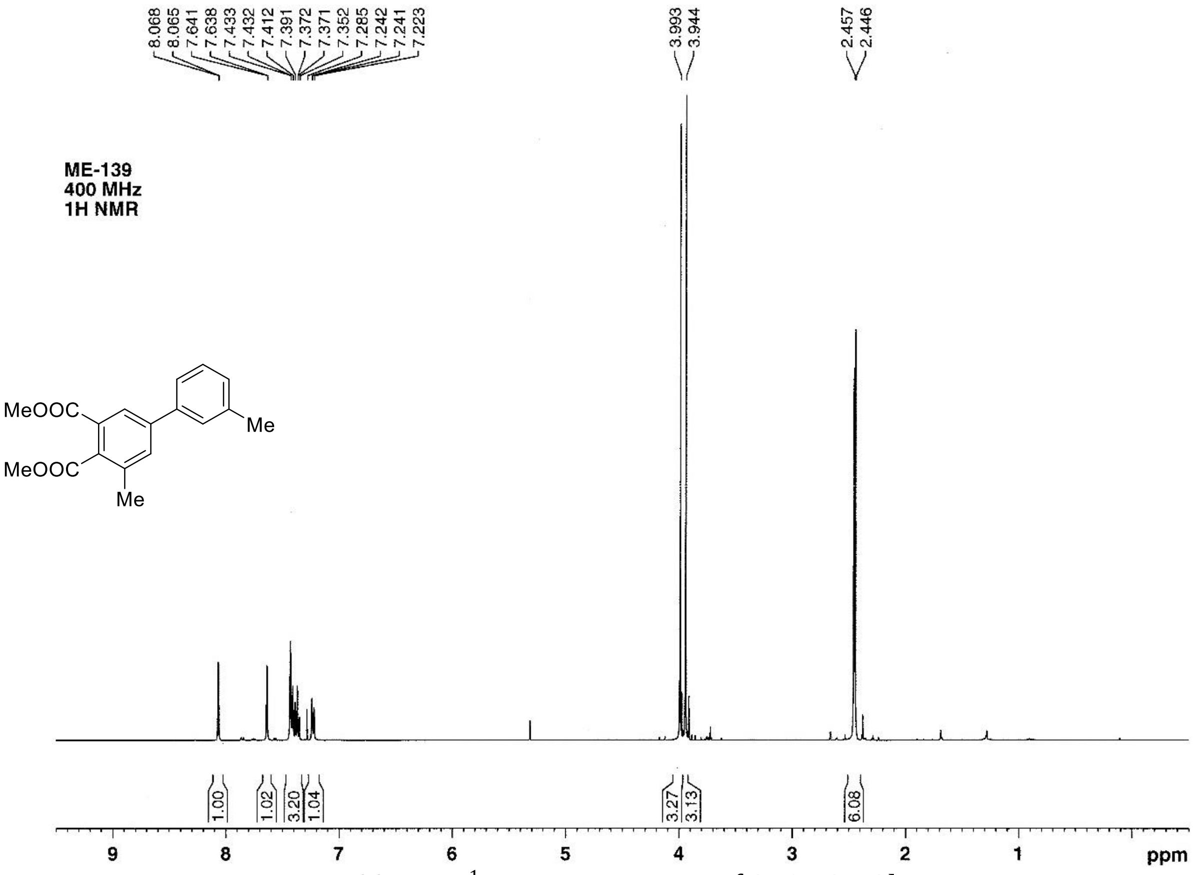

400 MHz ^{1}H NMR spectrum of **3e** in $CDCl_3$

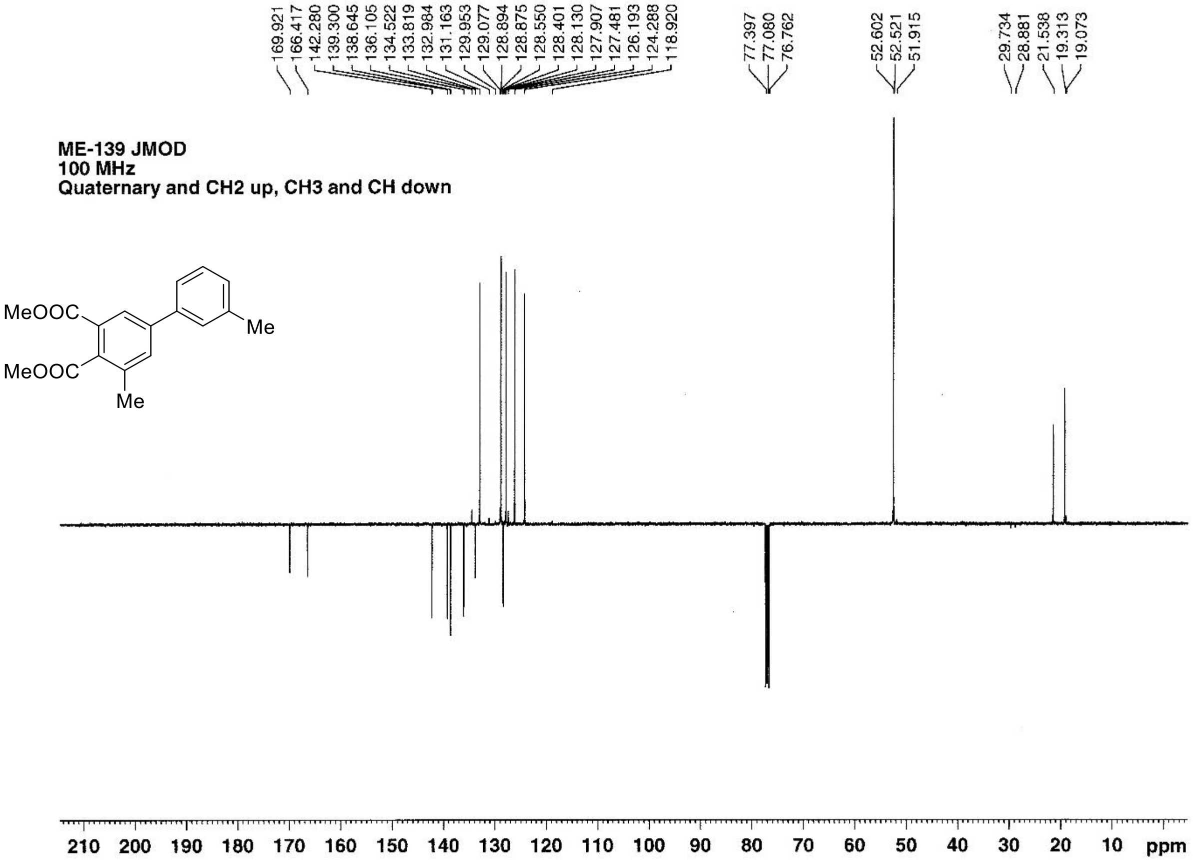

100 MHz ^{13}C NMR spectrum of **3e** in $CDCl_3$

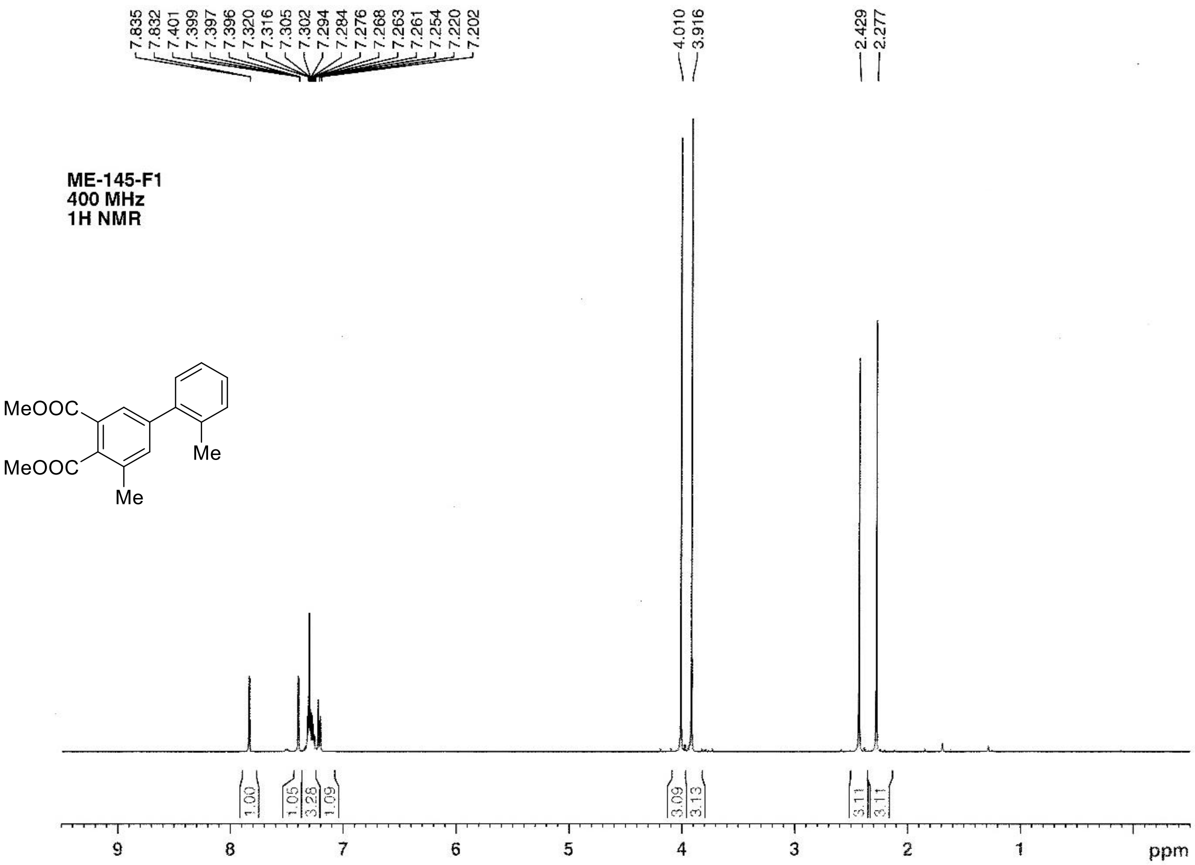

400 MHz ^{1}H NMR spectrum of **3f** in $CDCl_3$

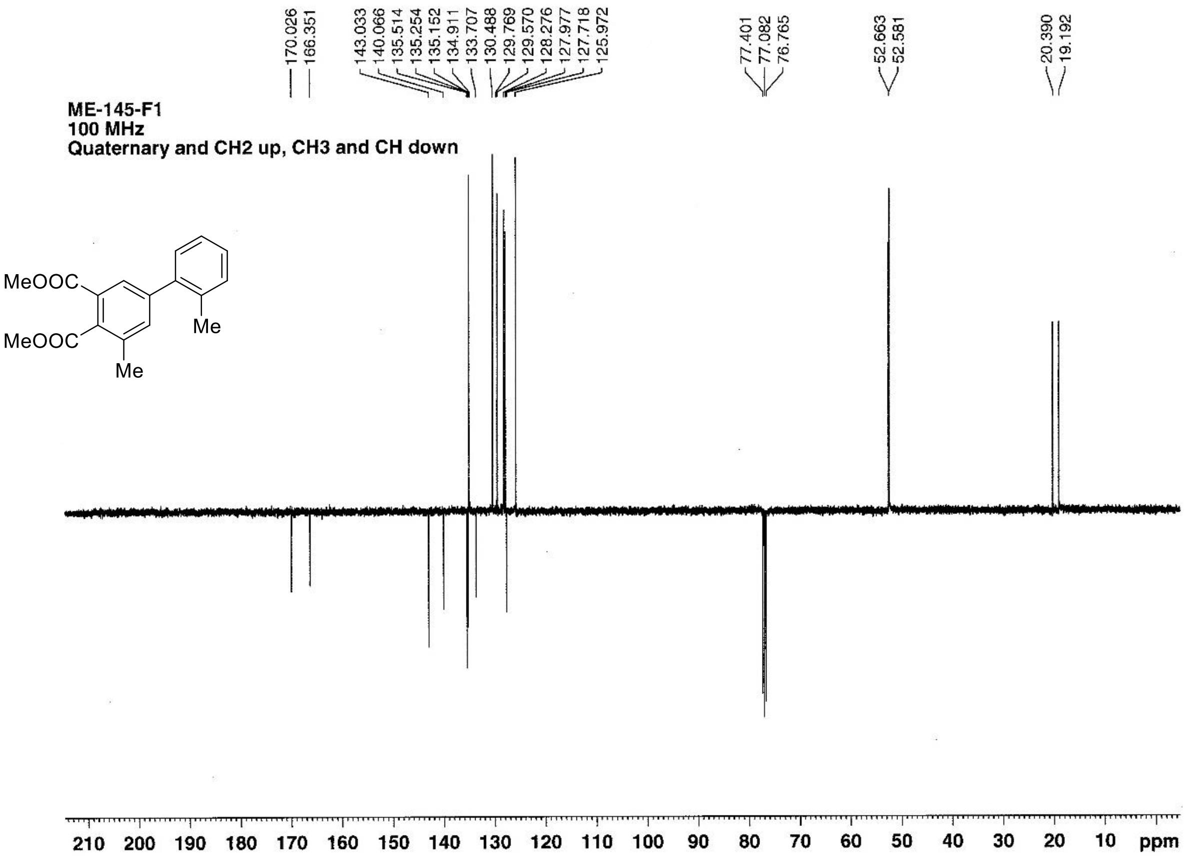

100 MHz ^{13}C NMR spectrum of **3f** in $CDCl_3$

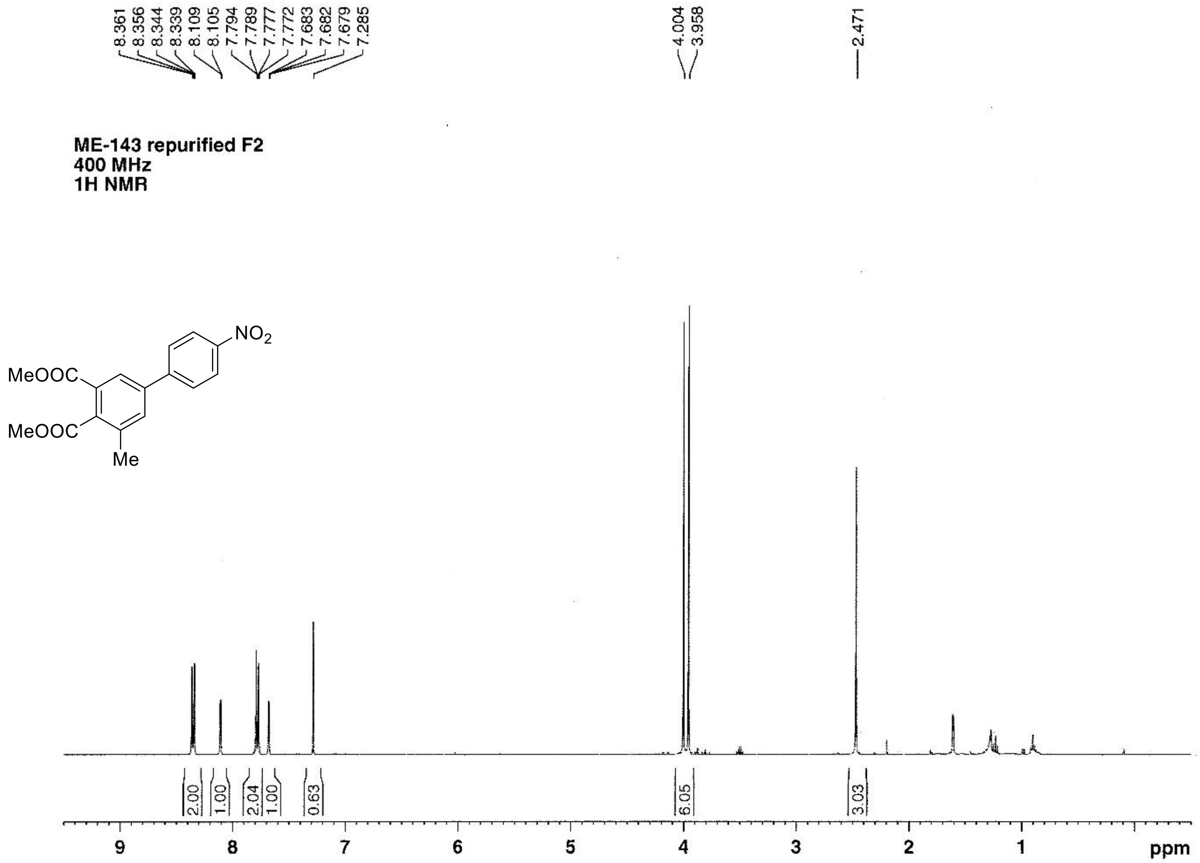

400 MHz ^{1}H NMR spectrum of **3g** in $CDCl_3$

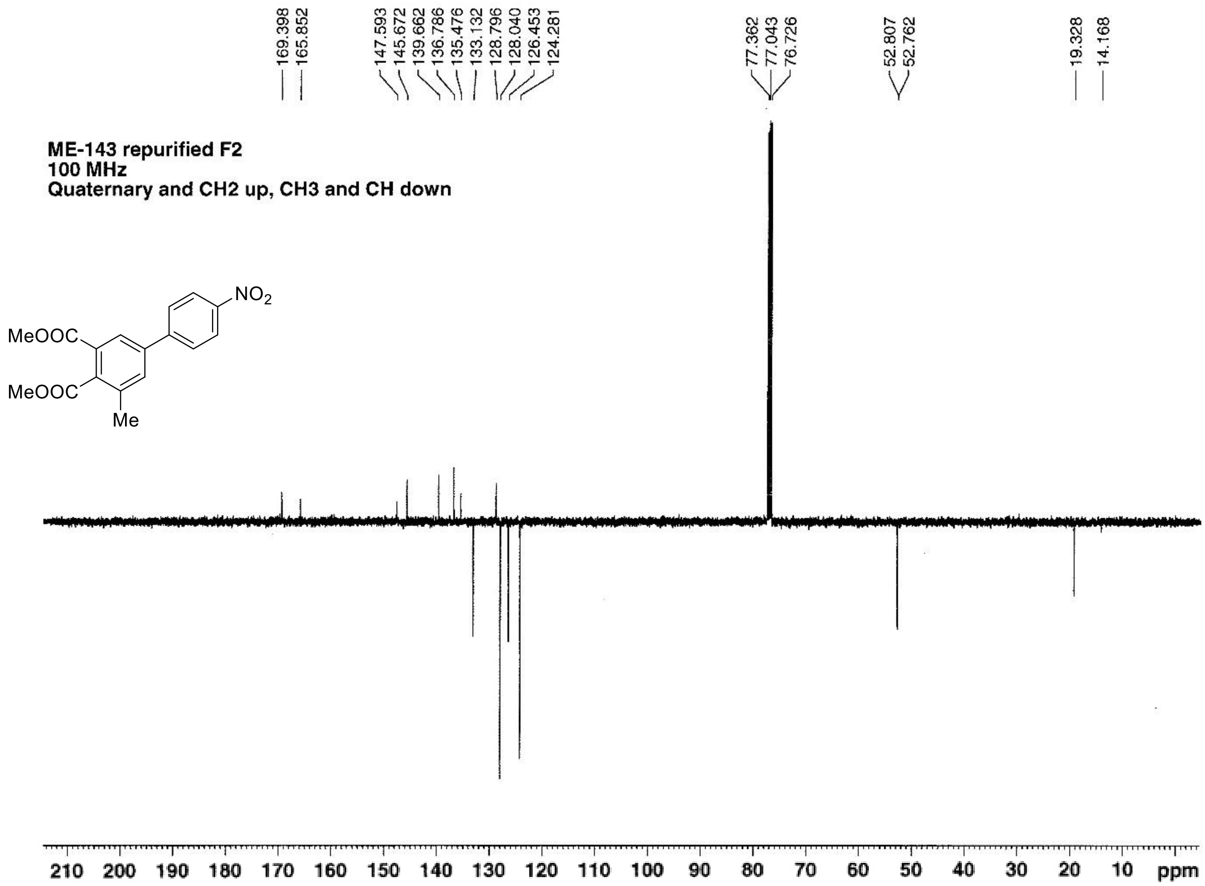

100 MHz ^{13}C NMR spectrum of **3g** in $CDCl_3$

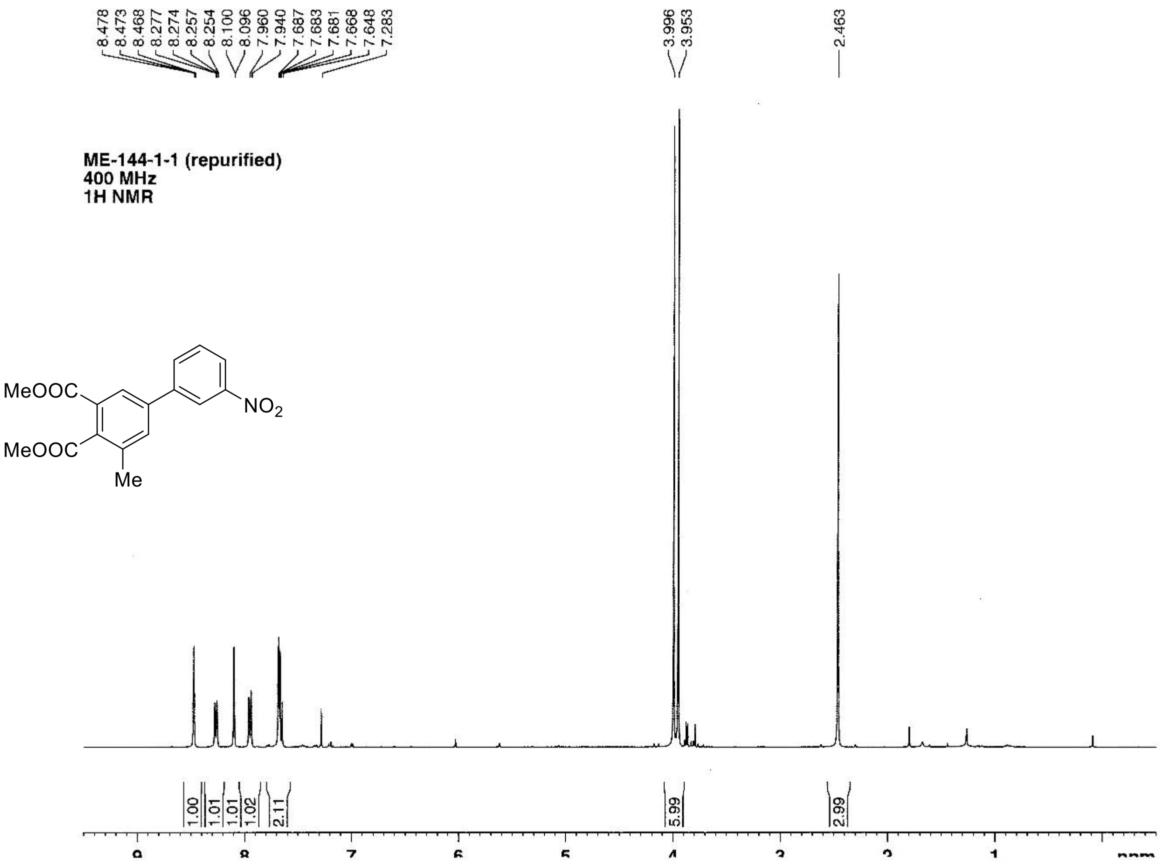

400 MHz ^{1}H NMR spectrum of **3h** in $CDCl_3$

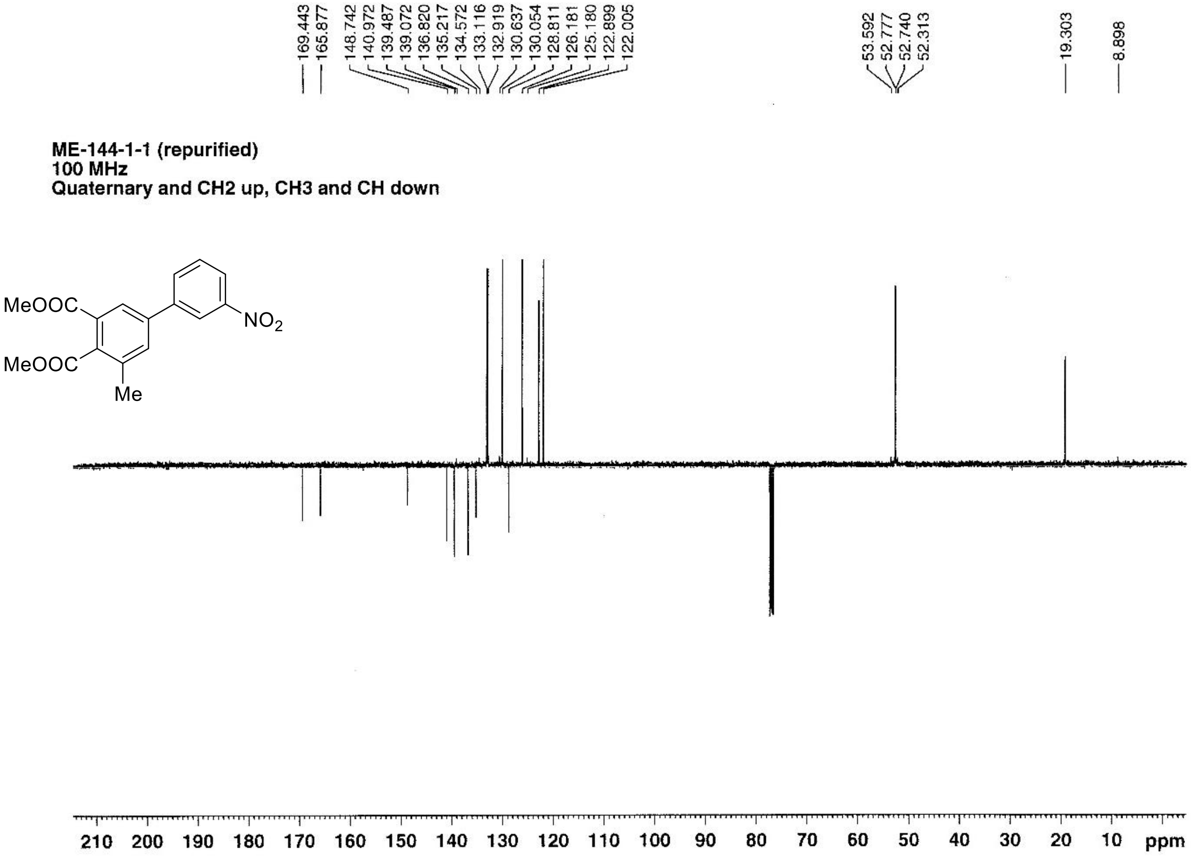

100 MHz ^{13}C NMR spectrum of **3h** in $CDCl_3$

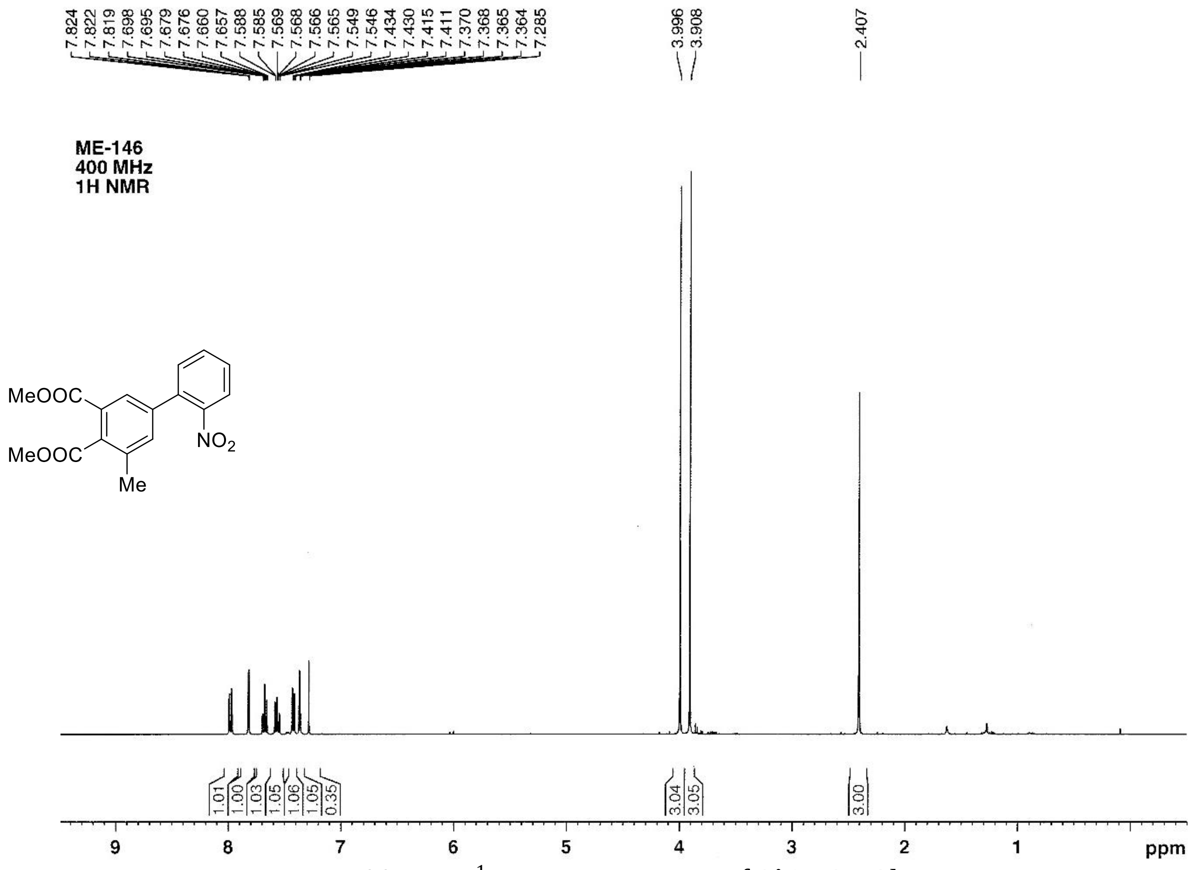

400 MHz ^{1}H NMR spectrum of **3i** in $CDCl_3$

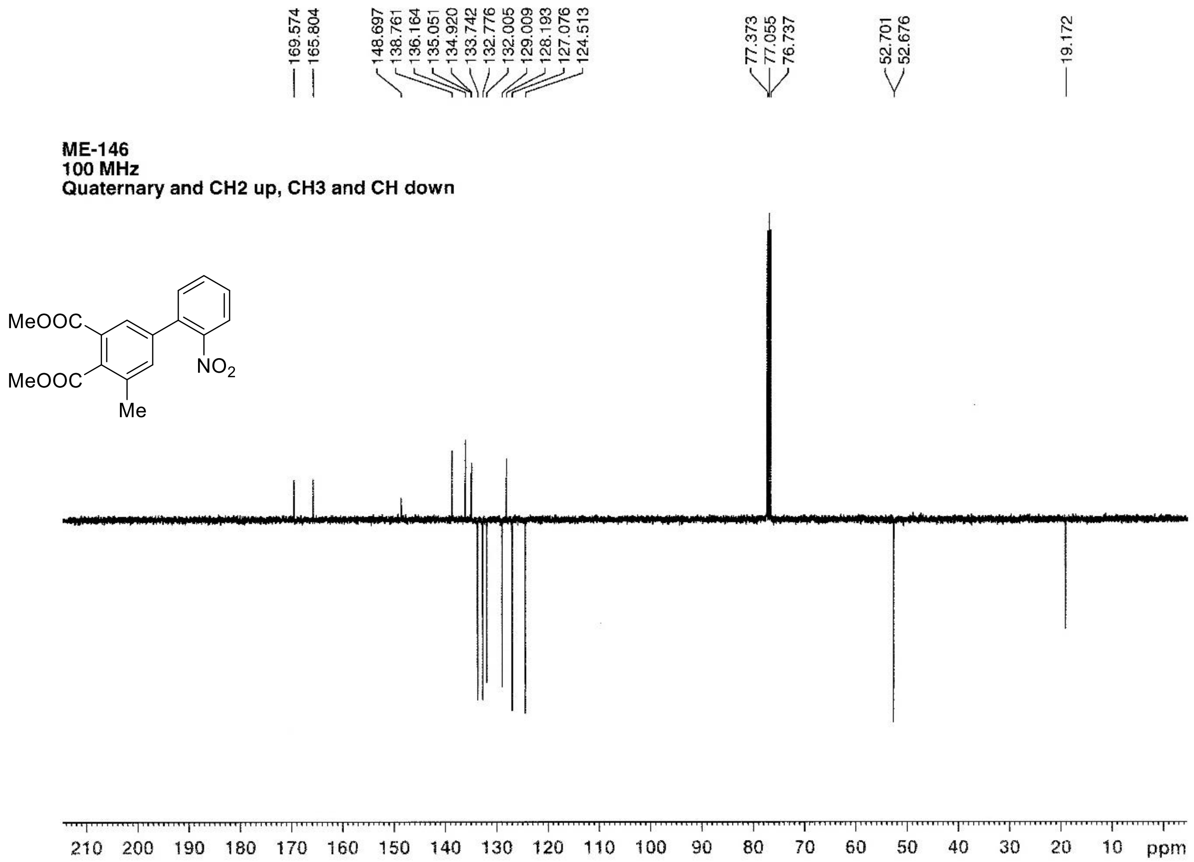

100 MHz ^{13}C NMR spectrum of **3i** in $CDCl_3$

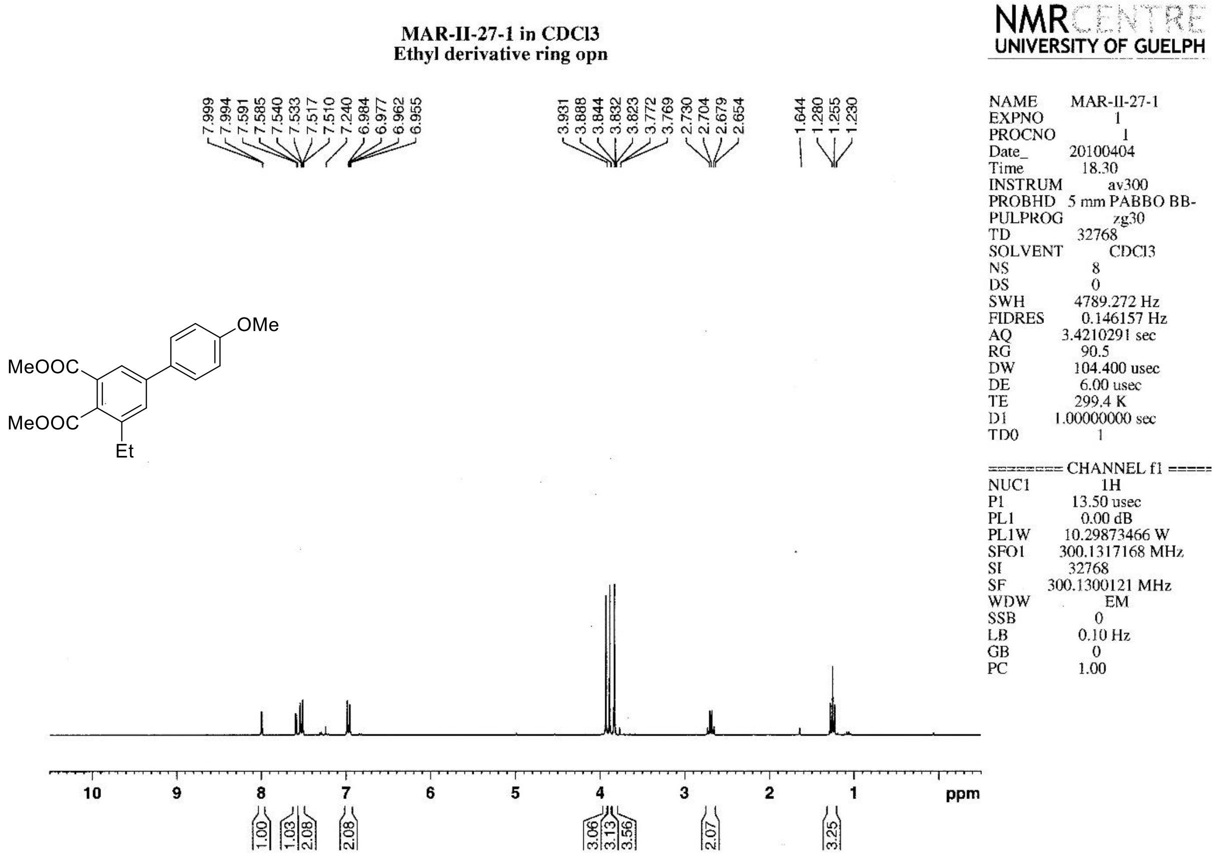

300 MHz ^{1}H NMR spectrum of **3j** in $CDCl_3$

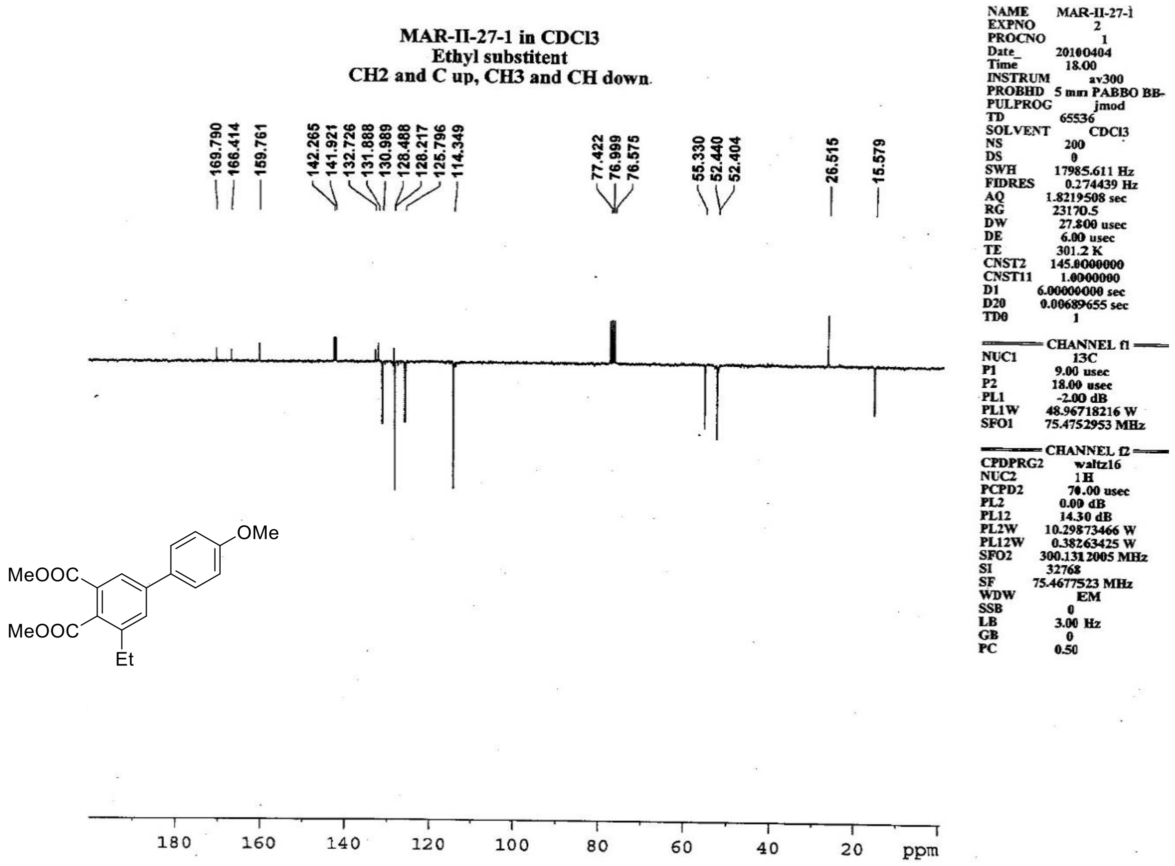

100 MHz ^{13}C NMR spectrum of **3j** in $CDCl_3$

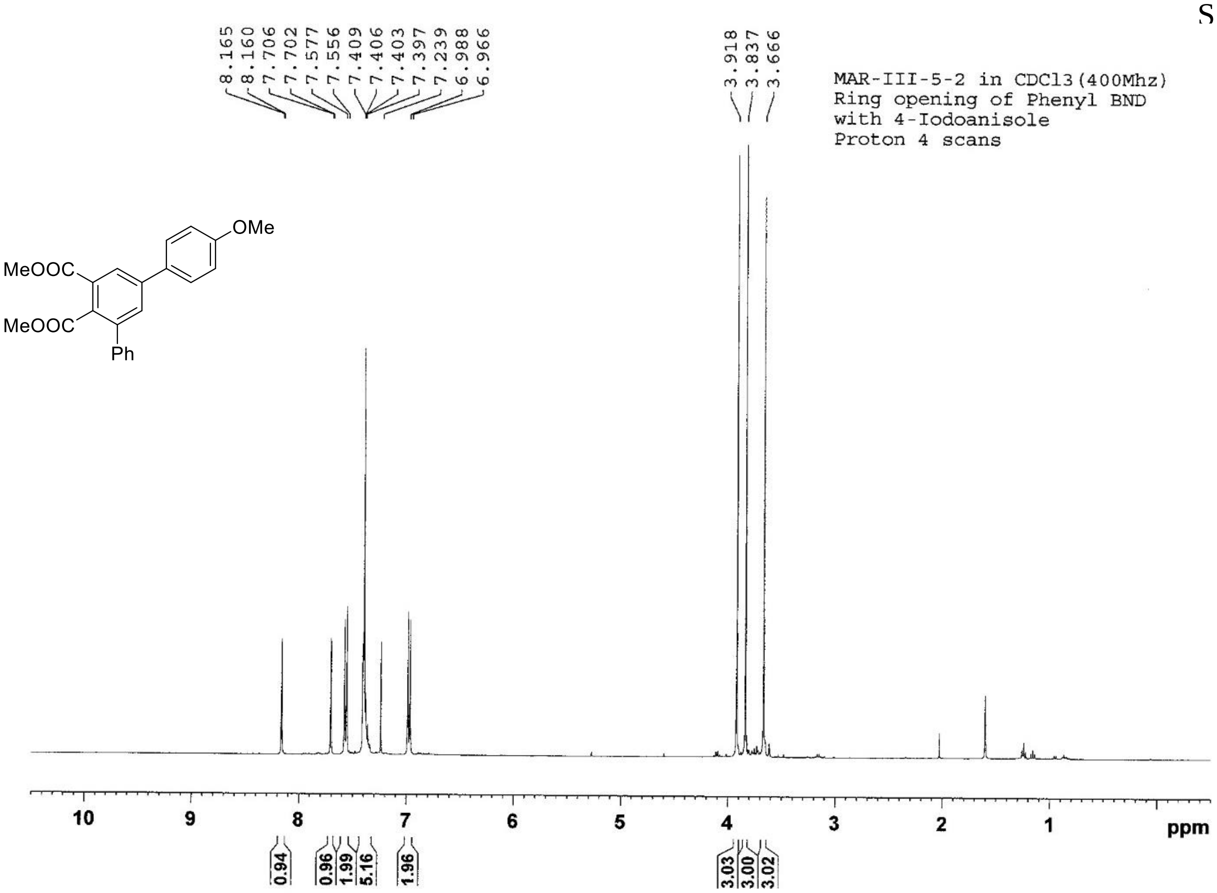

400 MHz ^{1}H NMR spectrum of **3k** in $CDCl_3$

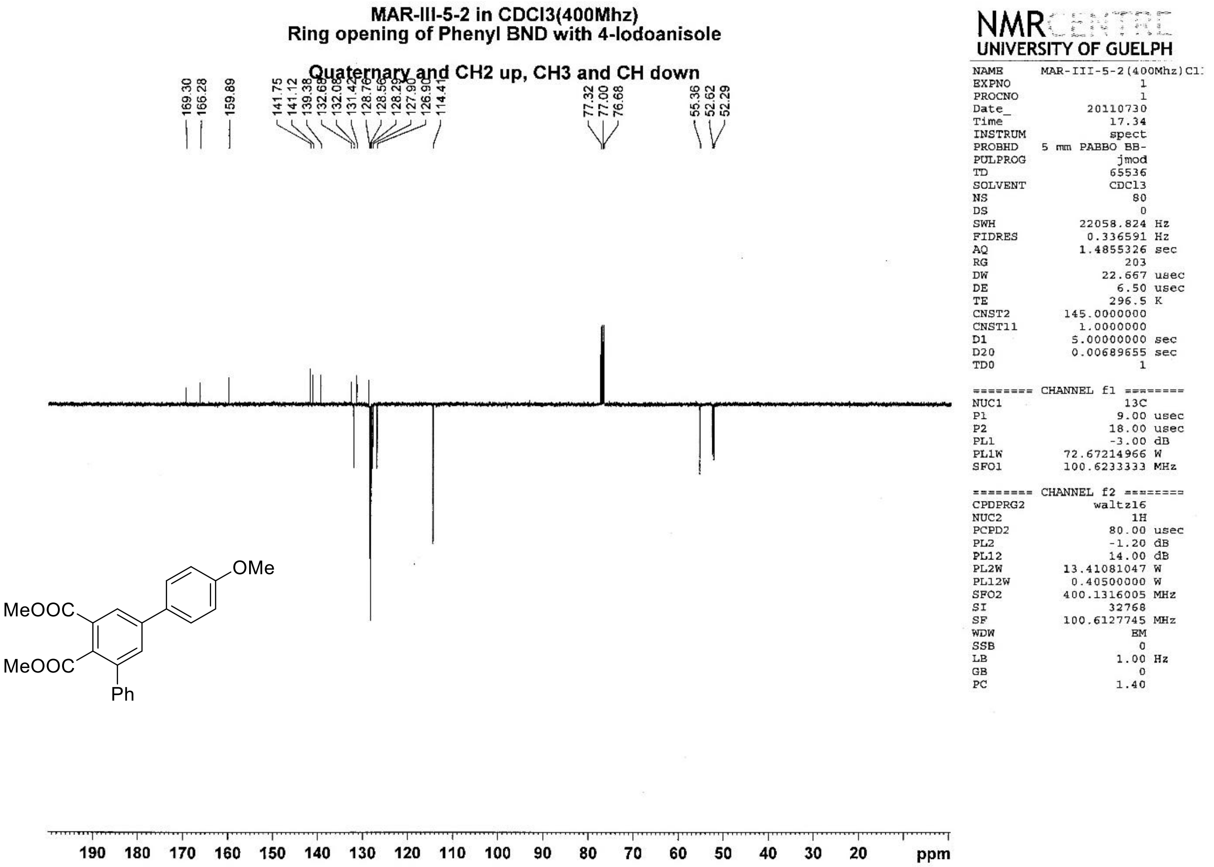

100 MHz ^{13}C NMR spectrum of **3k** in $CDCl_3$

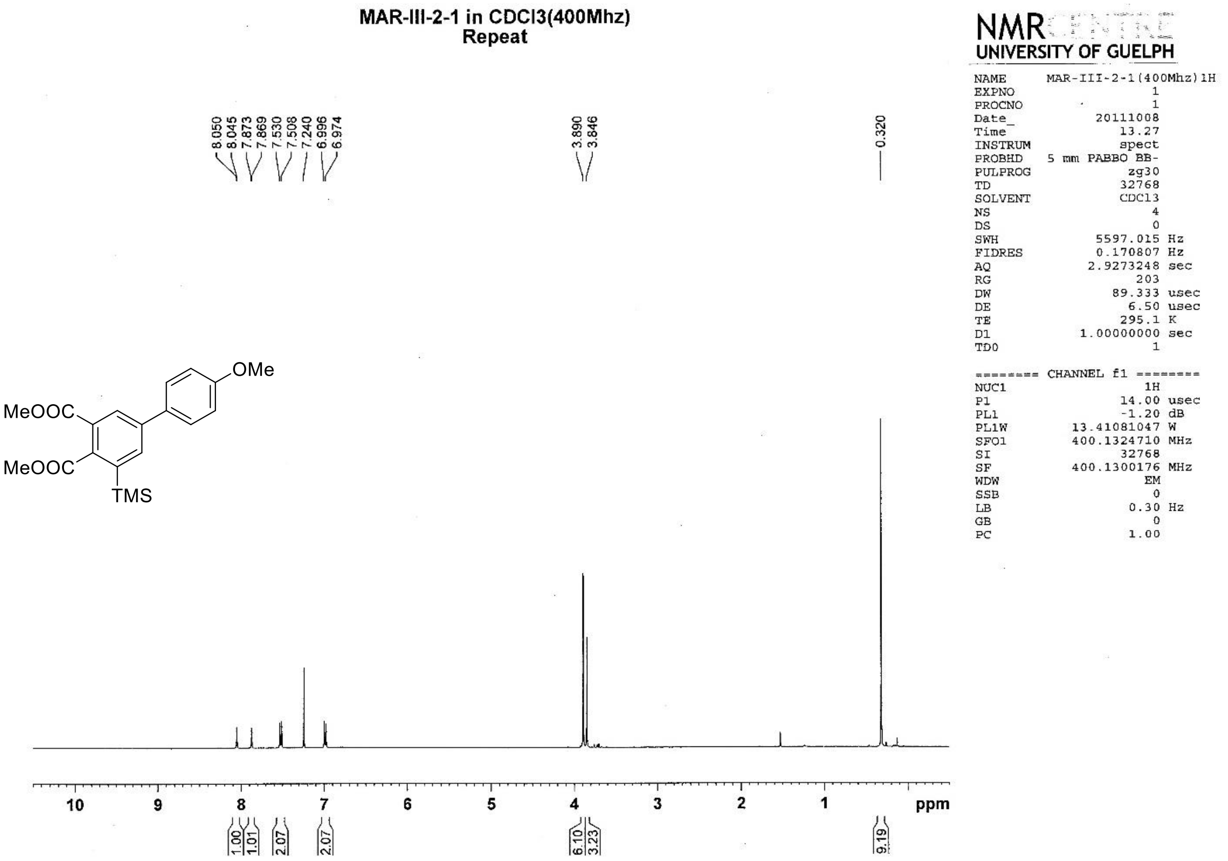

400 MHz ^{1}H NMR spectrum of **3l** in $CDCl_3$

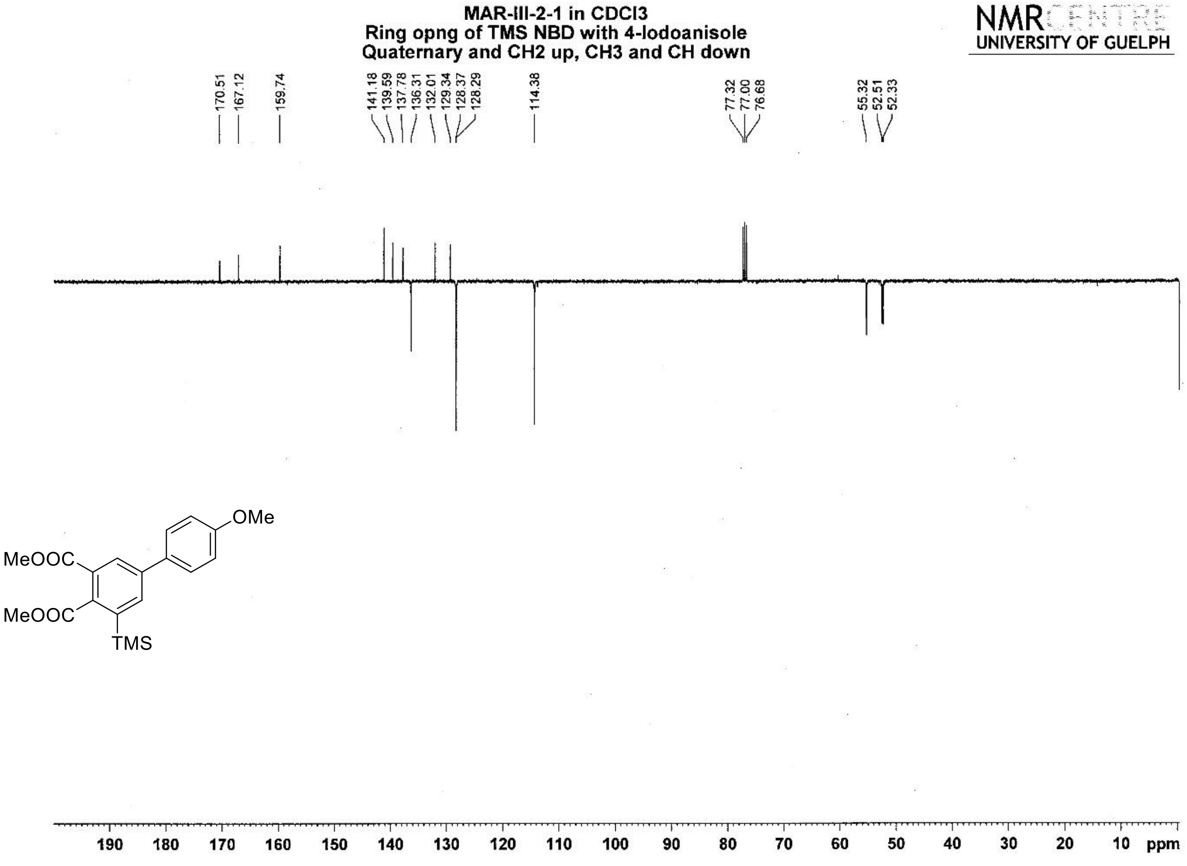

100 MHz ^{13}C NMR spectrum of **3l** in $CDCl_3$
